# Differential peripheral immune signatures elicited by vegan versus ketogenic diets in humans

**DOI:** 10.1038/s41591-023-02761-2

**Published:** 2024-01-30

**Authors:** Verena M. Link, Poorani Subramanian, Foo Cheung, Kyu Lee Han, Apollo Stacy, Liang Chi, Brian A. Sellers, Galina Koroleva, Amber B. Courville, Shreni Mistry, Andrew Burns, Richard Apps, Kevin D. Hall, Yasmine Belkaid

**Affiliations:** 1 Metaorganism Immunity Section, Laboratory of Host Immunity and Microbiome, National Institute of Allergy and Infectious Diseases, National Institutes of Health, Bethesda, MD USA; 2 NIH Center for Human Immunology, National Institutes of Health, Bethesda, MD USA; 3 Bioinformatics and Computational Biosciences Branch, Office of Cyber Infrastructure and Computational Biology, National Institute of Allergy and Infectious Diseases, National Institutes of Health, Bethesda, MD USA; 4 Center for Cellular Engineering, Department of Transfusion Medicine, Clinical Center, National Institutes of Health, Bethesda, MD USA; 5 Department of Cardiovascular and Metabolic Sciences, Lerner Research Institute, Cleveland Clinic, Cleveland, OH USA; 6 National Institute of Diabetes and Digestive and Kidney Diseases, National Institutes of Health, Bethesda, MD USA; 7 NIAID Microbiome Program, National Institute of Allergy and Infectious Diseases, National Institutes of Health, Bethesda, MD USA

**Keywords:** Feeding behaviour, Applied immunology, Metabolism, Data integration

## Abstract

Nutrition has broad impacts on all physiological processes. However, how nutrition affects human immunity remains largely unknown. Here we explored the impact of a dietary intervention on both immunity and the microbiota by performing a post hoc analysis of a clinical trial in which each of the 20 participants sequentially consumed vegan or ketogenic diets for 2 weeks (NCT03878108). Using a multiomics approach including multidimensional flow cytometry, transcriptomic, proteomic, metabolomic and metagenomic datasets, we assessed the impact of each diet, and dietary switch, on host immunity and the microbiota. Our data revealed that overall, a ketogenic diet was associated with a significant upregulation of pathways and enrichment in cells associated with the adaptive immune system. In contrast, a vegan diet had a significant impact on the innate immune system, including upregulation of pathways associated with antiviral immunity. Both diets significantly and differentially impacted the microbiome and host-associated amino acid metabolism, with a strong downregulation of most microbial pathways following ketogenic diet compared with baseline and vegan diet. Despite the diversity of participants, we also observed a tightly connected network between datasets driven by compounds associated with amino acids, lipids and the immune system. Collectively, this work demonstrates that in diverse participants 2 weeks of controlled dietary intervention is sufficient to significantly and divergently impact host immunity, which could have implications for precision nutritional interventions. ClinicalTrials.gov registration: NCT03878108.

## Abstract (web summary)

In a post hoc multiomics analysis of a trial comparing a plant-based, low-fat diet to an animal-based, ketogenic diet, the diets have differential effects on peripheral immune cell frequencies and immune-related gene expression.

## Main

Nutrition affects all physiological processes, including those that regulate our immune system^1^. The link between nutrition and host immunity represents an important opportunity to develop therapeutic nutritional interventions in the context of various disease states, such as cancer or chronic inflammatory disorders. In support of a link between diet and disease state, a low-fat vegan or vegetarian diet has been previously associated with decreased inflammation, reduced risk for cardiovascular diseases and reduction in overall mortality^2–4^. On the other hand, high-fat, very low-carbohydrate diets (commonly referred to as ketogenic diets) have been associated with reduced symptoms in defined types of epilepsy and reduced neuroinflammation^5–14^. However, despite the preventive and therapeutic potential of nutritional interventions, how nutrition impacts human immunity remains largely unknown.

Nutrition can impact host physiology via the amount and quality of fuels but also via the microbiota^15,16^. The microbiota possesses the ability to reconfigure and alter its function in ways that are believed to promote host resilience. As such, nutrition plays a dominant role in shaping the composition and function of the microbiome^17–23^. While the connection between the microbiota and nutrition is clearly established in experimental models, how such a symbiotic dyad influences human immunity remains largely unexplored.

In addition to the paucity of data pertaining to the impact of nutritional intervention on the human immune system, previous studies have explored responses to only one diet at a time. Based on the highly variable responses of individuals to nutritional interventions^24^ and the high number of diets consumed, addressing how individuals respond to different diets remains an important line of research. Moving forward, in the absence of rigorously designed clinical interventions, harnessing nutrition to shape human health will remain an ongoing challenge.

Here, we explored the impact of dietary interventions on both immunity and the microbiota in a highly controlled clinical setting, with each participant sequentially consuming distinct diets for 2 weeks in random order. To our knowledge, this study represents the first multiomics study investigating the impact of ketogenic and vegan diets on humans. Collectively, our results demonstrate a striking remodeling of host immunity and the microbiome and uncovered a divergent impact of ketogenic versus vegan diet. The insights derived from this work may have the potential to improve our understanding of diet-based therapeutic options for the prevention and treatment of disease.

## Dietary intervention alters lymphoid composition

**Fig. 1:**
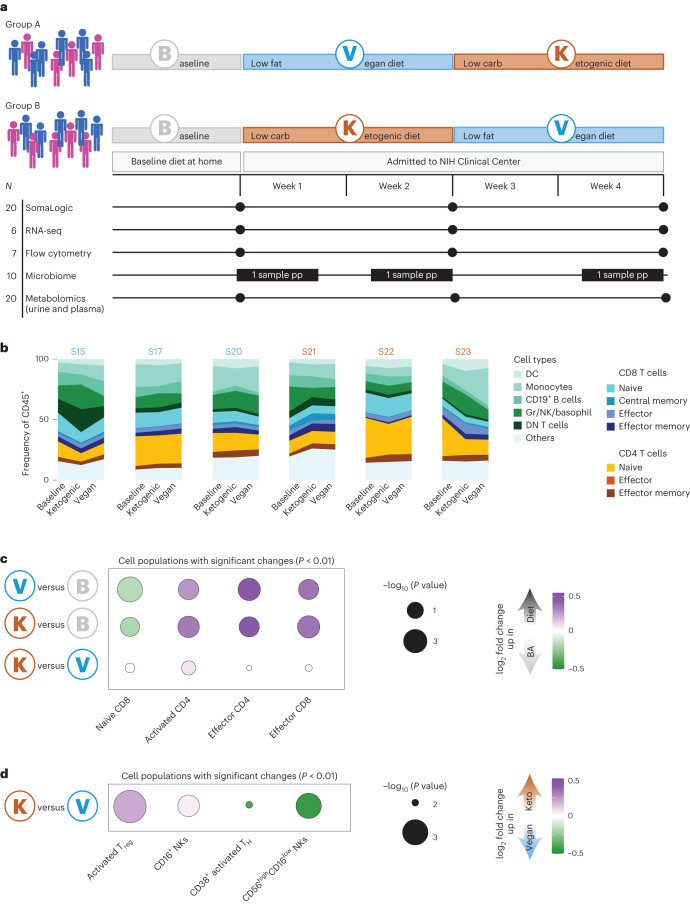
NK and T cells are significantly affected by change in diet. **a**, Schematic of experimental setup. Twenty participants were split into two groups (first group: 4 females (pink), 6 males (blue); second group: 5 females, 5 males), with one group starting on vegan diet for 2 weeks and then immediately changing to ketogenic diet (Group A), whereas the other group started with ketogenic diet and changed to vegan diet (Group B). Data (indicated on the bottom) were collected directly before first diet as baseline, and at the end of the first and second diets. For microbiome samples, data were collected on different days (refer to Extended Data Fig. 1k for more details). **b**, Frequency of main cell types (as frequency of all CD45^+^ live cells) measured by flow cytometry for baseline, ketogenic and vegan diets shown for each participant. For the gating strategy for flow cytometry, see Extended Data Table 1 and Extended Data Fig. 2. Order of diet listed in this panel is the same for all participants independent of their first diet. Color of individual on top of the plot denotes starting diet (orange, ketogenic diet; blue, vegan diet). **c**, Fold changes of cell populations whose frequency significantly changed between ketogenic/vegan diet and baseline diet (*P* value < 0.01) (purple, upregulated in vegan/ketogenic diet; green, upregulated in baseline diet). Dots are scaled by −log_10_(*P* value). Significance was calculated by two-sided paired *t*-test. **d**, Fold change of cell populations whose frequency significantly changed between ketogenic and vegan diets (*P* value < 0.01) (purple, upregulated in ketogenic diet; green, upregulated in vegan diet). Dots are scaled by −log_10_(*P* value). Significance was calculated by two-sided paired *t*-test. For gating strategy refer to Extended Data Fig. 2 and ref. ^37^. Regulatory T (T_reg_) cells, CD127^low^CD25^high^CCR4^+^HLA-DR^+^; CD16^+^ NK cells, CD3^−^CD19^−^CD14^−^HLA-DR^−^CD123^−^CD56^+^CD16^+^; activated T helper (T_H_) cells, CD3^+^CD19^−^CD4^+^CD8^−^HLA-DR^+^CD38^+^; activated NK cells, CD3^−^CD19^−^CD14^−^HLA-DR^−^CD123^−^CD56^+^CD16^low^CD57^high^. BA, baseline; DC, dendritic cells; DN, double negative; Gr, granulocytes; pp, per person; S, sample.

**Extended Data Fig. 1:**
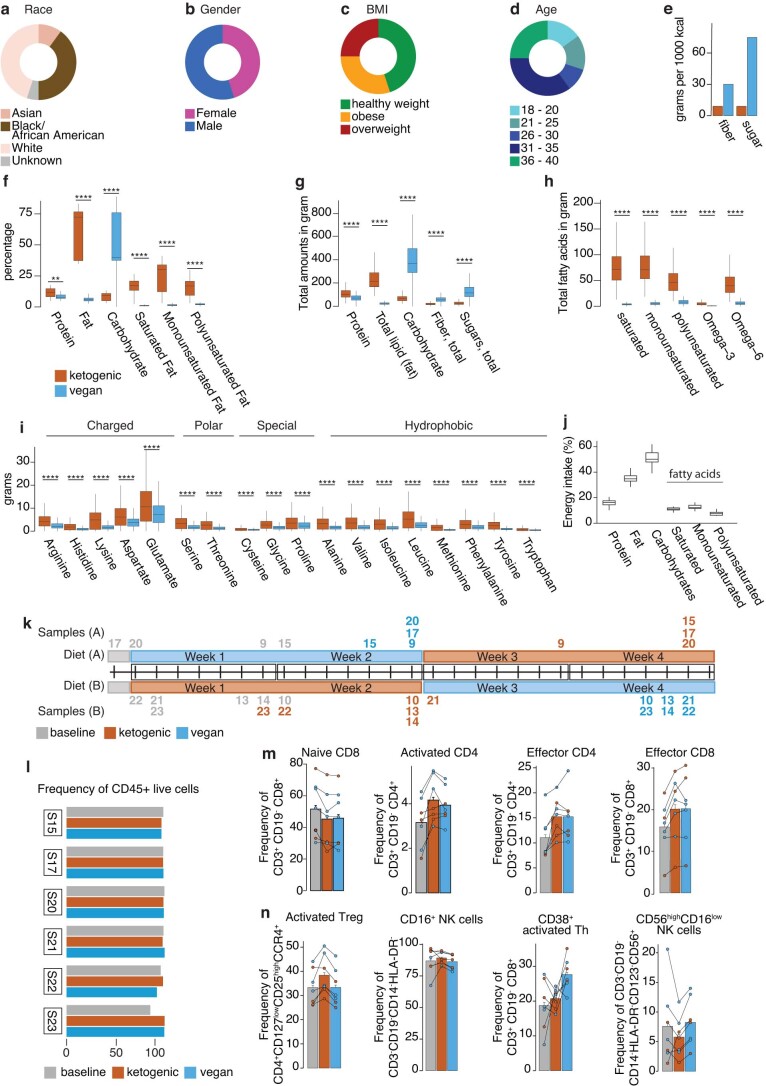
Background information about study cohort and diets. (**a–d**) Distribution of different characteristics in the study population. Plots showing race (**a**), gender (**b**), body mass index (BMI) (**c**), and age (**d**). (**e**) Amount of dietary fiber and dietary sugar in ketogenic and vegan diet in grams per 1000 kcal. (**f, g**) Box-whisker plot showing amounts of consumed components of diets in percentage (**f**) and total grams (**g**). (**h**) Amount of consumed types of fatty acid in diets in grams. (**i**) Amount of consumed amino acids in diets in grams. Significance was calculated by two-sided paired t-test with multiple testing correction. (**j**) Percentage of energy intake from different nutrients at baseline based on food questionnaire analysis. (**k**) Schematic showing when microbiome data was collected. (**l**) Frequency of CD45^+^ live cells in flow cytometry data. (**m**) Bar graph of frequencies of all cell types significantly different between baseline and diet (from Fig. 1c). Each dot represents one individual. Dots are colored by starting diet (blue: vegan, orange: ketogenic). (**n**) Bar graph of frequencies of all cell types significantly different between ketogenic and vegan diet (from Fig. 1d). Each dot represents one individual. Dots are colored by starting diet (blue: vegan, orange: ketogenic). Data is represented as box-whisker plots in **f-j**. The lower and upper hinges of the box correspond to the first and third quartiles (the 25th and 75th percentiles), the line in the box indicate the median. The upper whisker extends from the hinge to the largest value no further than 1.5 × interquartile range (IQR) from the hinge and the lower whisker extends from the hinge to the smallest value at most 1.5 × IQR of the hinge mean (n = 20). Data is represented as bar graphs in **m,n**. Error bars show standard deviation (n = 7). P-values: * < 0.05; ** < 0.01; *** < 0.001; **** < 0.0001.

We performed a highly controlled nutritional study in 20 participants admitted to the National Institutes of Health (NIH) Clinical Center (Fig. 1a). In this cross-over study, a diverse cohort of participants (Extended Data Fig. 1a–d) consumed ad libitum a ketogenic, low-carbohydrate diet (75.8% fat, 10.0% carbohydrate) and a vegan, low-fat diet (10.3% fat, 75.2% carbohydrate) for 2 weeks at random, and in different orders (Fig. 1a). Both diets had a common foundation of nonstarchy vegetables (~1 kg per day) with low amounts of digestible carbohydrates and only minimum amounts of highly processed food. The ketogenic diet added animal-based products including meat, poultry, fish, eggs, dairy and nuts. The vegan diet added legumes, rice, root vegetables, soy products, corn, lentils, peas, whole grains, bread and fruit. The vegan diet was high in dietary fiber and dietary sugars as compared with the ketogenic diet (Extended Data Fig. 1e). The nutrient intake of participants between the two diets differed significantly in their composition (Extended Data Fig. 1f,g). Further, participants on ketogenic diet consumed higher amounts of fatty acids and amino acids (Extended Data Fig. 1h,i). Details about the ketogenic and vegan diets, including photographs of the presented meals, were previously published^25^.

Baseline food intake was estimated with the help of a food questionnaire (Extended Data Fig. 1j). A previous report based on this cohort confirmed increased ketone bodies in participants consuming a ketogenic diet and demonstrated that participants on a vegan diet consumed significantly fewer calories compared with those on a ketogenic diet^25^. Blood samples were collected at several time points and cell population composition was assessed via flow cytometry (*n* = 7), gene expression via bulk RNA sequencing (RNA-seq) (*n* = 6) and protein composition via SomaLogic (*n* = 20). Fecal samples were collected for microbiome metagenomic sequencing (*n* = 10) (Extended Data Fig. 1k) and metabolomic analysis was performed on both blood and urine (*n* = 20). Of note, because of sample availability not all assays could be performed on all participants.

**Extended Data Fig. 2:**
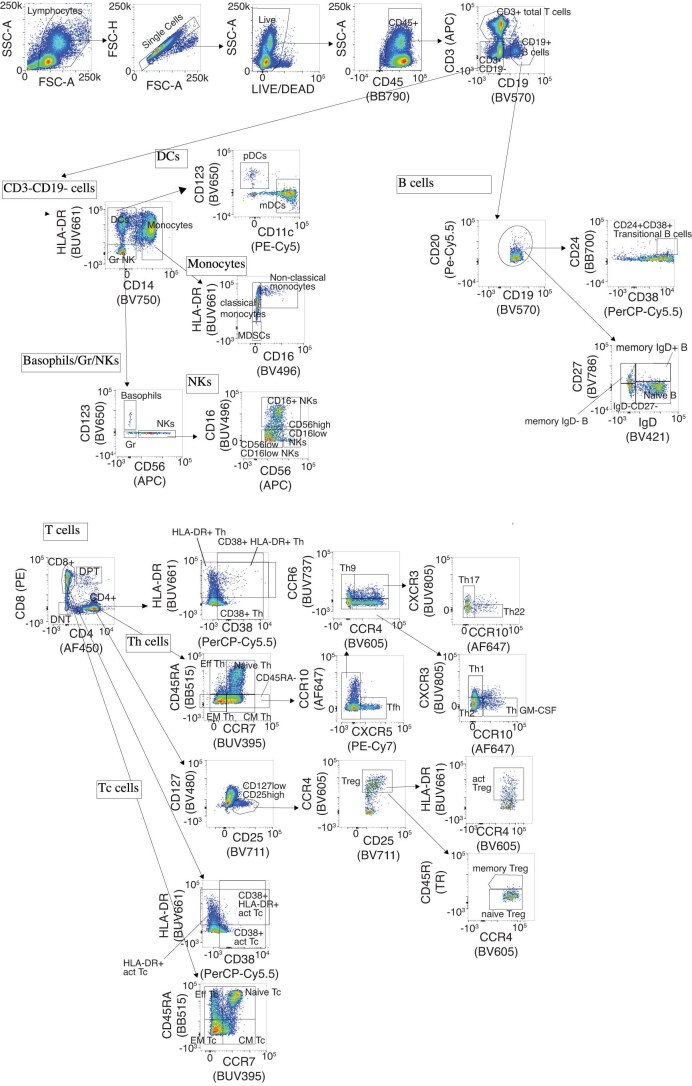
**Gating strategy for high-dimensional flow cytometry data**.

We first assessed the cellular composition of peripheral blood mononuclear cells (PBMCs) via flow cytometry (Extended Data Figs. 1l and 2, Extended Data Tables 1 and 2 and ref. ^26^). PBMC analysis focused on all major immune cell types, except for neutrophils which do not survive sample processing. As expected, high variability was observed at baseline between participants (for example, frequency of naive CD4 T cells ranged from 5% of all CD45^+^ cells to almost 25%) (Fig. 1b).

Notably, change in diet itself, independent of diet order, induced significant changes, including a significant decrease in the level of naive CD8 T cells and a significant increase in the level of activated CD4 T cells, effector CD4 T cells and effector CD8 T cells following both ketogenic/vegan versus baseline diet (Fig. 1c and Extended Data Fig. 1m). Whether these changes resulted from the shift in diet, or an abrupt decrease in the consumption of highly processed food which is often represented in a standard Western diet, remains unclear but would be of interest for future investigation.

Some distinct changes were also observed following consumption of each diet, independent of diet order. For instance, we observed a significant increase in the frequency of activated regulatory T cells and CD16^+^ natural killer (NK) cells following consumption of a ketogenic diet compared with vegan diet (Fig. 1d and Extended Data Fig. 1n). Further, we observed a significant increase in the frequency of activated T helper cells and activated NK cells following vegan diet compared with ketogenic diet (Fig. 1d and Extended Data Fig. 1n). Thus, changes in diet itself had a significant impact on the host immune system. Further, both ketogenic and vegan diets imposed distinct changes in lymphoid composition and status of activation.

## Vegan and ketogenic diets impose divergent immune signatures

**Extended Data Fig. 3:**
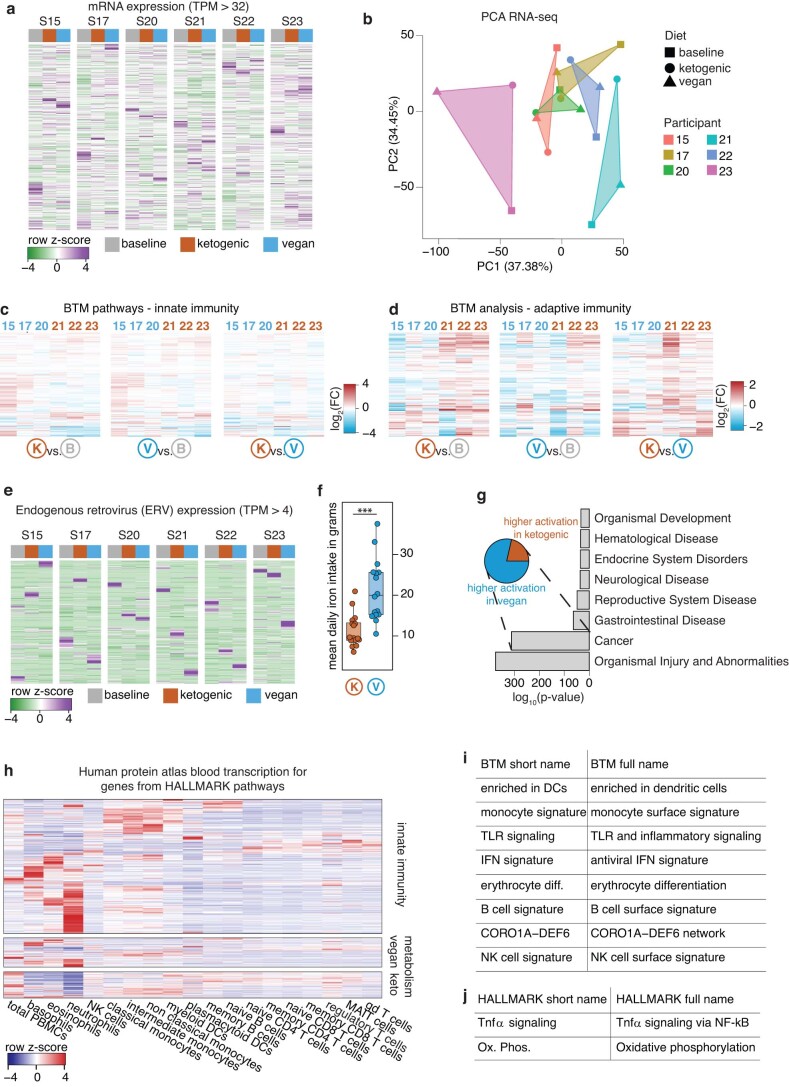
Supporting data for RNA-seq analysis. (**a**) Heat map of all expressed genes (TPM > 32) in all participants and diets. (**b**) Principal component analysis (PCA) of RNA-seq data. (**c**) Heat map showing log 2 foldchange of all genes from significant innate immunity pathways (BTM analysis). Participant number is denoted on top of heat map and colored by first diet (blue: vegan, orange: ketogenic). (**d**) Heat map showing log 2 foldchange of all genes from significant adaptive immunity pathways (BTM analysis). Participant number is denoted on top of heat map and colored by first diet (blue: vegan, orange: ketogenic). (**e**) Heat map of all expressed endogenous retroviruses (ERVs) (TPM > 4). (**f**) Mean total daily iron intake in grams in ketogenic and vegan diet. Data is represented as box-whisker plot. The lower and upper hinges of the box correspond to the first and third quartiles (the 25th and 75th percentiles), the line in the box indicate the median. The upper whisker extends from the hinge to the largest value no further than 1.5 × interquartile range (IQR) from the hinge and the lower whisker extends from the hinge to the smallest value at most 1.5 × IQR of the hinge (n = 20). Significance was calculated by a two-sided paired t-test. (**g**) Bar graph showing the number of pathways significantly enriched in IPA disease term analysis comparing ketogenic diet with vegan diet. Pie chart shows proportion of cancer pathways with stronger activation following ketogenic diet (orange) and vegan diet (blue). (**h**) Heat map of gene expression (as row Z-score) from sorted populations from the blood downloaded from the Human Protein Atlas^35^. Depicted genes are members of pathways significantly differentially enriched between ketogenic and vegan diet in HALLMARK analysis. (**i**) Table of BTM long names – referred to Fig. 2a (**j**) Table of HALLMARK long names – referred to Fig. 2b. P-values: *** < 0.001.

We next performed bulk RNA-seq of whole blood at baseline and following diets. Clustering of highly expressed genes showed marked differences in the expression of transcripts between all three conditions, as well as between individuals (Extended Data Fig. 3a). Principal component analysis (PCA) showed that principal component 1 (PC1) captured differences in the transcriptome between participants explaining 37.38% of variation, whereas PC2 captured differences between the diets explaining 34.45% of variation (Extended Data Fig. 3b). As expected, most variations resulted from interindividual differences; however, diet also had a significant impact on whole blood transcriptome.

**Fig. 2:**
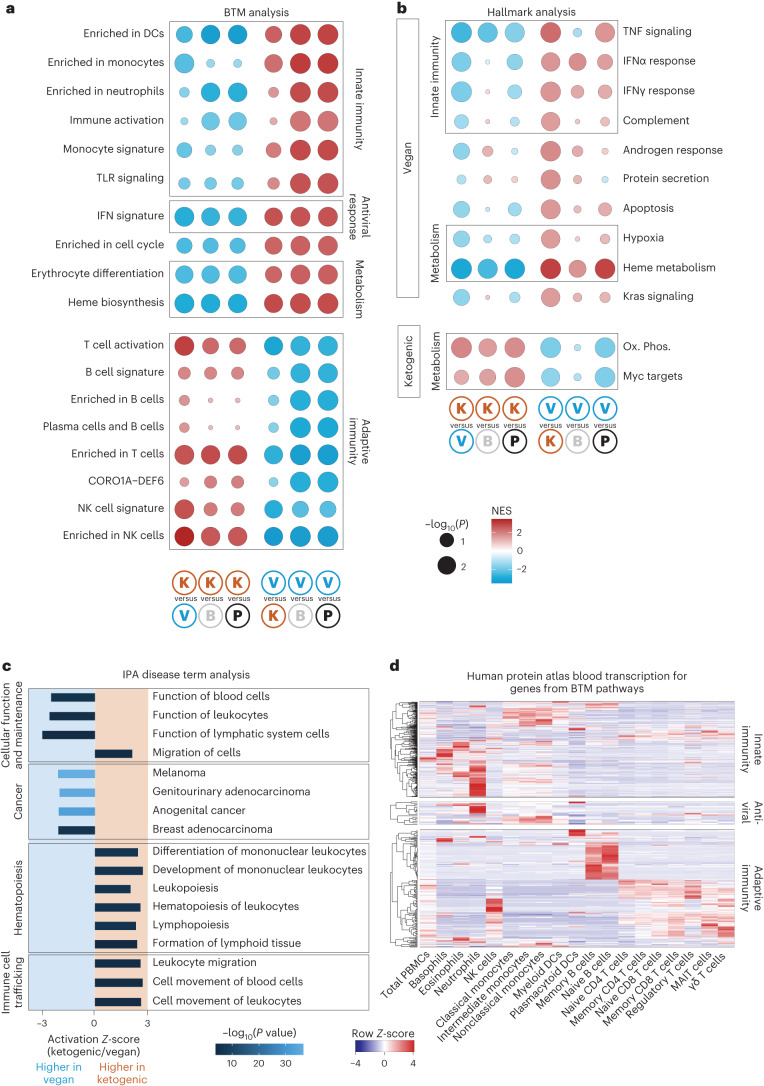
Ketogenic diet is associated with heightened adaptive immunity and vegan diet with heightened innate immunity. **a**, BTM analysis showing enriched pathways for all comparisons noted on the bottom. Dots are scaled by −log_10_(*P* value) and colored by network enrichment score (NES). Category names were shortened. Refer to Extended Data Fig. 3i for full names. Significance was calculated and multiple-testing corrected with the fgsea pathway package. **b**, Hallmark analysis showing enriched pathways for all comparisons noted on the bottom. Dots are scaled by −log_10_(*P* value) and colored by NES. Category names were shortened. Refer to Extended Data Fig. 3j for full names. Significance was calculated and multiple-testing corrected with the fgsea pathway package. **c**, Bar graph showing results from IPA disease term analysis. The *x* axis shows activation *Z*-score (comparing ketogenic versus vegan diet). Bars are colored by −log_10_(*P* value). Positive *Z*-score values show disease terms enriched in ketogenic diet, whereas negative *Z*-scores show disease terms enriched in vegan diet. Significance was calculated using Fisher’s exact test. **d**, Heat map of gene expression (as row *Z*-score) from sorted populations from the blood downloaded from the Human Protein Atlas^35^. Depicted genes are members of pathways significantly differentially enriched between ketogenic and vegan diets in BTM analysis. B, baseline; K, ketogenic; V, vegan; P, previous diet.

We next assessed functional trajectories associated with each diet. To this end, we performed blood transcription module^27^ (BTM) analysis as well as Hallmark analysis of all genes differentially expressed between each diet comparison (Fig. 2a,b and Extended Data Fig. 3c,d), with previous diet referring to the diet consumed directly beforehand (see Methods for more details). This approach uncovered a striking polarization in overall pathway enrichment between ketogenic and vegan diets (Fig. 2a,b). For example, ketogenic diet was associated with an upregulation of pathways linked to adaptive immunity, including T cell activation and enrichment of B cells and plasma cells, as well as NK cells (Fig. 2a and Extended Data Fig. 3d). As such, oxidative phosphorylation, a fundamental pathway associated with T cell activation and memory formation (reviewed in refs. ^28–30^), was significantly enriched in ketogenic diet compared with vegan or baseline diet (Fig. 2b).

In contrast, a vegan diet was associated with upregulation of pathways associated with innate immunity, as well as antiviral responses (Fig. 2a,b and Extended Data Fig. 3c). Functional analysis further predicted upregulation of type I interferon signatures and responses (Fig. 2a,b). The order of diets did not affect transcriptional changes (Extended Data Fig. 3c,d). We and others have shown that sensing of endogenous retroviruses (ERVs) can contribute to host immunity and that changes in dietary lipids impact ERV expression^31–33^. Indeed, we observed distinct changes in ERV expression both between individuals and after dietary changes, where discrete sets of ERVs were uniquely upregulated in each participant after defined diets (Extended Data Fig. 3e).

Based on study design, the overall daily intake of dietary iron, an important component of erythropoiesis, was higher in vegan compared with ketogenic diet (Extended Data Fig. 3f). Accordingly, we observed an upregulation of erythrocyte differentiation as well as heme biosynthesis and metabolism in vegan diet only. To attempt to correlate diet-associated signature with disease states, we next performed Ingenuity Pathway Analysis (IPA) disease term analysis (Fig. 2c). This analysis confirmed our BTM and Hallmark analyses, with an increase in red blood cell-associated pathways following vegan diet and increased lymphopoiesis following ketogenic diet. We also saw higher activation of pathways associated with cancer in vegan compared with ketogenic diet. A total of 308 cancer-associated pathways were significant, of which 66 were predicted to have higher activation following ketogenic diet, and 242 were predicted to show stronger activation following vegan diet (Extended Data Fig. 3g). Four pathways reached an activation score difference of 2 or greater, all of which showed stronger activation following vegan diet (Fig. 2c). Of note, these observations alone do not predict any differences in patients’ susceptibility to cancer and cancer outcome. Our data suggest that both ketogenic and vegan diets might influence cancer outcome, and preliminary evidence supports the idea that ketogenic diet might be beneficial in conjunction with other cancer treatments^34^, whereas there are no published studies investigating the impact of vegan diet on cancer. However in-depth epidemiologic studies in humans, and mechanistic studies in animal models, would be required to validate these potential associations and their link to beneficial or worsening outcomes.

To predict drivers of transcriptional changes, we evaluated the expression profiles of sorted cell populations from the blood^35^ and analyzed gene expression from enriched pathways. Using this approach, we found that upregulation of innate immunity following vegan diet was predicted to be driven mainly by neutrophils, whereas upregulation of adaptive immunity in ketogenic diet was predicted to be driven by B and T cells (Fig. 2d and Extended Data Fig. 3h).

Overall, our data highlighted a divergent effect of diet on the immune system, with ketogenic diet enriching for adaptive immune signatures and vegan diet enriching for innate immune signatures.

## Ketogenic diet has a broader impact on the proteosome

**Fig. 3:**
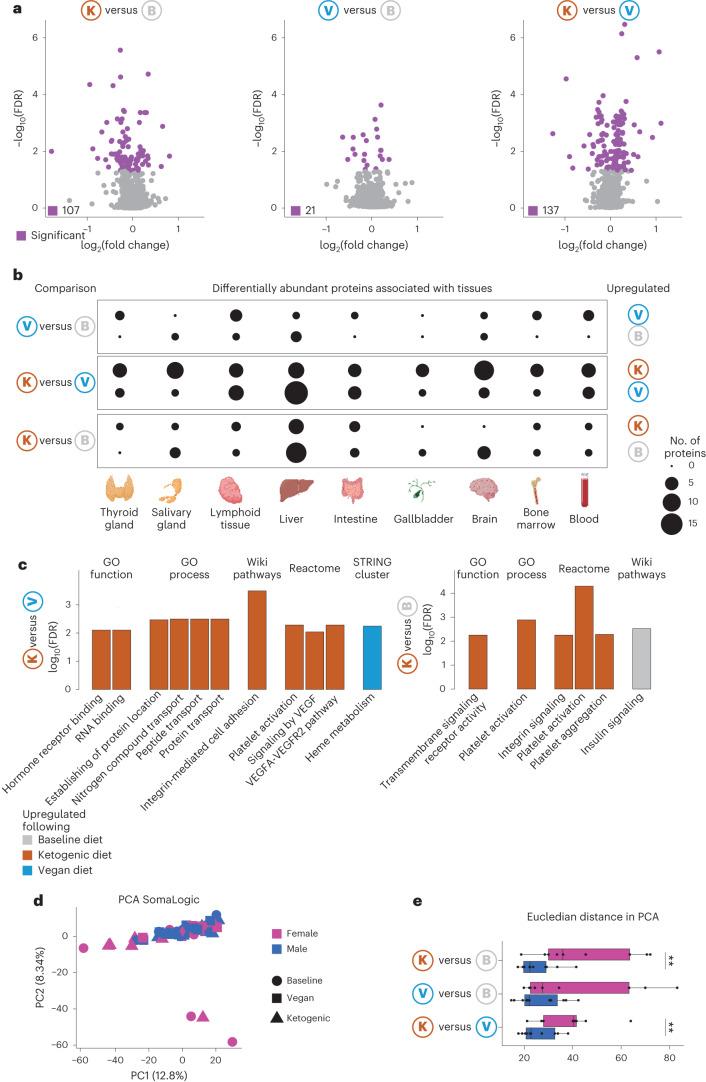
Proteomics data show upregulation of adaptive immunity following ketogenic diet. **a**, Volcano plot for protein abundance of comparisons noted on top. Proteins that are significantly different (fold change greater than 2, false discovery rate (FDR) < 0.01) are colored purple. Significance was calculated with a paired Wilcoxon signed-rank test with multiple-testing correction. **b**, Dot plot showing tissue origin of differentially abundant proteins. Dots are scaled by number of proteins. **c**, Bar graph showing functional enrichment analysis using STRING. Analysis was performed on fold changes of all proteins between ketogenic and vegan (left) and ketogenic and baseline diets (right). Orange bars denote upregulation in ketogenic diet, blue in vegan, gray in baseline diet. **d**, PCA of proteome data colored by sex. **e**, Box-and-whisker plot showing Euclidean distance from PCA separated by sex. The lower and upper hinges of the box correspond to the first and third quartiles (the 25th and 75th percentiles); the line in the box indicates the median. The upper whisker extends from the hinge to the largest value no further than 1.5 × interquartile range (IQR) from the hinge and the lower whisker extends from the hinge to the smallest value at most 1.5 × IQR from the hinge (*n* = 20, 11 males/9 females). Significance was calculated by a paired two-sided *t*-test. **P < 0.01. GO, gene ontology; FDR, false discovery rate.

**Extended Data Fig. 4:**
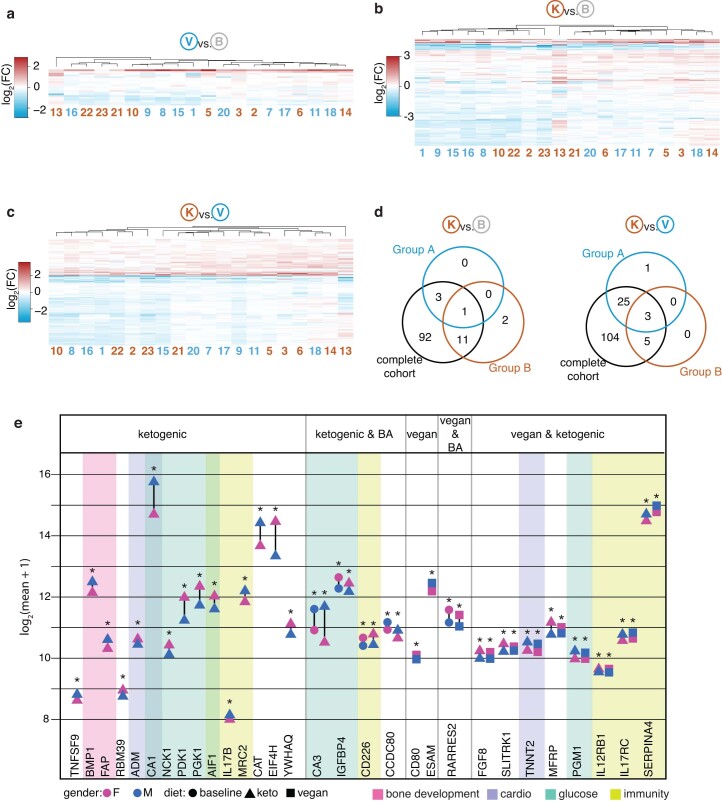
Supporting data for proteomics data. (**a-c**) Heat map of log2 fold change with participants colored by starting diet (blue: vegan, orange: ketogenic) for all significant differentially abundant proteins for vegan diet versus baseline diet (**a**), ketogenic diet versus baseline diet (**b**), and ketogenic diet versus vegan diet (**c**). (**d**) Venn diagram showing overlap of significantly differentially abundant proteins from complete data set, data from group A (starting diet: vegan), and group B (starting diet: ketogenic) for ketogenic diet versus baseline diet (left) and ketogenic diet versus vegan diet (right). There were no significantly differentially abundant proteins for baseline diet versus vegan diet in group A and group B. (**e**) Proteins that are significantly differentially abundant between female and male participants. Significance was calculated by Student’s t-test with multiple testing correction.

We next measured the abundance of about 1,300 proteins via SomaLogic from plasma of all 20 participants at baseline and post ketogenic or vegan diet. Applying a linear mixed effects model (LME) showed no significant difference between protein abundance in vegan and ketogenic diets between participants from different groups (*P* = 0.5624). Analysis of variance (ANOVA) revealed that a fraction of proteins were significantly impacted between diets. Notably, ketogenic diet had the largest impact on protein abundance (baseline versus ketogenic, 107; vegan versus ketogenic, 137), while only a few proteins were significantly altered between baseline and vegan diet (21) (Fig. 3a). Diet order did not affect direction or magnitude of fold change of differentially abundant proteins (Extended Data Fig. 4a–c). Additionally, ANOVA applied to data from participants with different starting diets did not reveal additional proteins significantly impacted by diets, confirming that diet order does not impact the effect of diet on protein abundance (Extended Data Fig. 4d).

We next analyzed the origin of differentially impacted proteins by downloading tissue annotations from the Human Protein Atlas^35^. Ketogenic diet impacted proteins predicted to originate from several tissues, including the blood, brain and bone marrow, while both diets affected proteins predicted to originate from the liver and secondary lymphoid organs (Fig. 3b). Thus, a ketogenic diet may have a broader impact on host protein secretion or clearance than a vegan diet.

We performed functional enrichment analysis with STRING^36^ based on fold change of all proteins (Fig. 3c). Consistent with results gained from transcriptomics analysis, we observed a significant enrichment in heme metabolism following vegan diet (Fig. 3c). Of note, we also observed an enrichment of insulin signaling pathway in baseline diet compared with ketogenic diet (Fig. 3c).

PCA did not show separation by diet but showed several outliers (Fig. 3d). Further analysis revealed that all outliers were female participants who showed substantially greater changes following ketogenic diet, highlighting potential sex-bias in responsiveness to diet (Fig. 3e). Sex-specific differences in protein abundance between diets included proteins associated with glucose metabolism, as well as immunity (Extended Data Fig. 4e).

Thus, proteomic data analysis revealed that a ketogenic diet may have the strongest effect on the proteome of study participants. Furthermore, proteomic data supported the idea that vegan diet can promote heme metabolism and that there is a sex-specific difference in the magnitude of response to diet.

## Ketogenic diet downregulates microbial amino acid metabolism

**Fig. 4:**
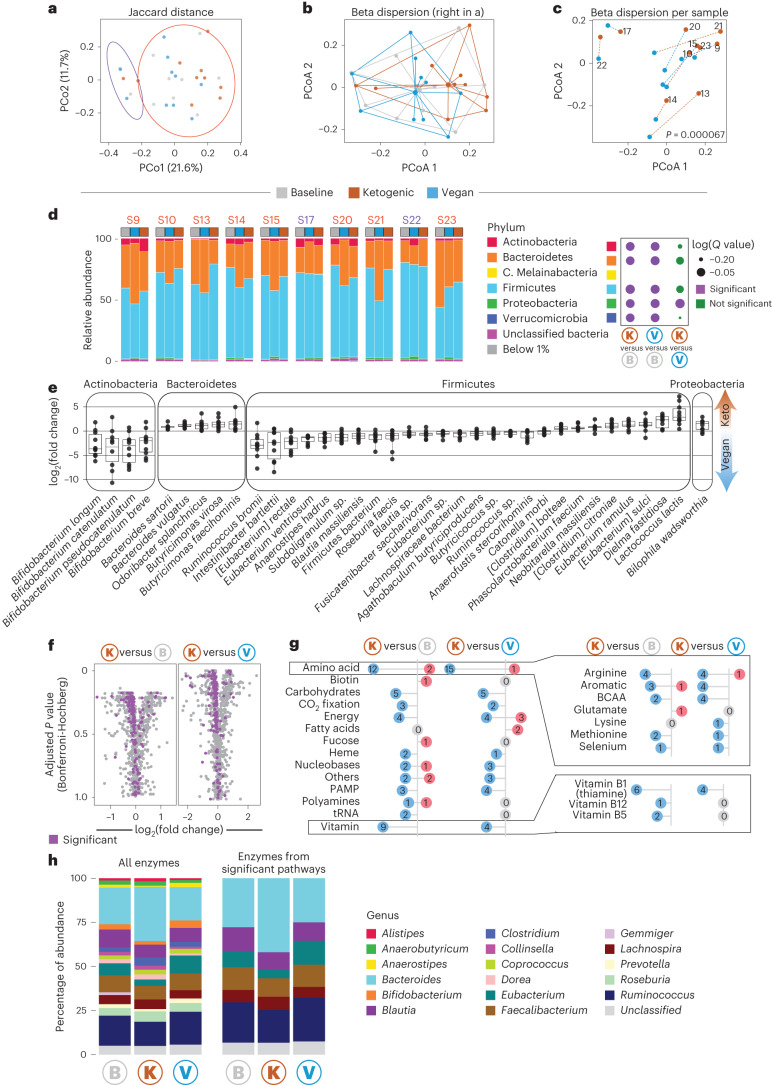
Ketogenic diet significantly alters composition and function of microbiome. **a**, PCoA of microbiome data with 95% confidence interval. The data split into two clusters. **b**, Centroid analysis for beta dispersion plot. **c**, Beta diversity plot for each individual from PCoA analysis with connection between ketogenic and vegan diets for each participant. Connection lines are colored by starting diet. Significance was calculated with a PERMANOVA test using a marginal model ~Diet + SubjectID. **d**, Stacked bar graph showing distribution of abundant phyla (>1%) for all individuals following baseline, ketogenic and vegan diets (left). Individuals on top are colored based on the cluster membership of panel **a**. Significance was calculated using Maaslin2 and *P* values were adjusted with the qvalue R package. Dot plot shows significance of changes in phylum between diet comparisons (right). Purple dots show significant changes, whereas green dots denote no significance. **e**, Box-and-whisker plot of fold change of significantly differentially abundant species between ketogenic and vegan diets for all significant taxa (*Q* value < 0.2). The lower and upper hinges of the box correspond to the first and third quartiles (the 25th and 75th percentiles); the line in the box indicates the median. The upper whisker extends from the hinge to the largest value no further than 1.5 × IQR from the hinge and the lower whisker extends from the hinge to the smallest value at most 1.5 × IQR from the hinge (*n* = 10). **f**, Volcano plot showing fold change of abundance of EC numbers for ketogenic diet versus baseline diet (left) and ketogenic diet versus vegan diet (right). Purple dots show enzymes from significantly differently abundant pathways for each comparison. Significance was calculated using Maaslin2 and *P* values were Bonferroni–Hochberg corrected. **g**, Lollipop plot showing number of significantly changed pathways from MetaCyc enrichment analysis (left) and changed subpathways for amino acids and vitamin biosynthesis (right) for ketogenic diet versus baseline diet and ketogenic diet versus vegan diet. **h**, Stacked bar graph showing which genera contribute to pool of all enzymes (left) and all enzymes from significantly differently enriched pathways between ketogenic diet and vegan diet (right). PAMP, pathogen-associated molecular patterns.

**Extended Data Fig. 5:**
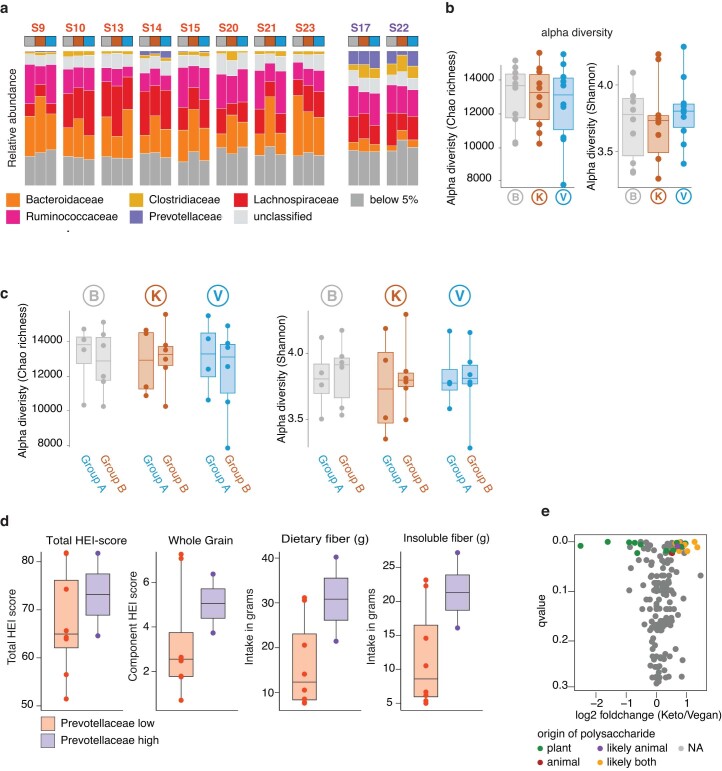
Supporting data for microbiome data. (**a**) Stacked bar graph showing differences between clusters from Fig. 4a which is driven by differences in abundance of *Prevotella*. Color on top denotes which cluster the data was from in Fig. 4a. S: Sample (**b**) Alpha diversity measured by Chao richness (left) and Shannon diversity (right). (**c**) Alpha diversity measured by Chao richness (left) and Shannon diversity (right) divided by group per diet. (**d**) Box-whisker plots showing overall health index (HEI) score and intake of fiber at baseline diet based on food questionnaire. **(e)** Volcano plot of microbial enzymes colored by source of polysaccharide degradation. Data is represented as box-whisker plots in **b - d**. The lower and upper hinges of the box correspond to the first and third quartiles (the 25^th^ and 75^th^ percentiles), the line in the box indicate the median. The upper whisker extends from the hinge to the largest value no further than 1.5 × interquartile range (IQR) from the hinge and the lower whisker extends from the hinge to the smallest value at most 1.5 × IQR of the hinge (n = 10 for **b, c**; in **d**: n = 8 for Prevotellaceae low, n = 2 for Prevotellaceae high).

Host diet is one of the main drivers of microbiota composition and function^17–23^. As such, we performed microbiome metagenomic sequencing, which allows analysis of changes in microbiota composition and predicted function. Principal coordinate analysis (PCoA) showed no clear separation between diets (Fig. 4a); however, data clustered in two different clusters. Further investigation showed that, in line with previous studies^37^, clusters were broadly characterized by high versus low *Prevotella* abundance before dietary intervention (Extended Data Fig. 5a). Such differences may have been driven by variations in fiber intake during baseline diet, although analysis of the food questionnaires intended to examine baseline diet did not show any significant differences in fiber intake in participants (Extended Data Fig. 5d).

While we did not see any significant differences in the diversity of the microbiome per participant between diets (alpha diversity), we saw significant differences in the microbiome composition between ketogenic and vegan diet (beta diversity), demonstrating a shift in microbiome composition following consumption of a ketogenic diet (Fig. 4b,c and Extended Data Fig. 5b). We did not find significant differences in alpha diversity (*P* = 0.1028) or beta diversity between groups per diet (*P* = 0.75952 for Shannon diversity, *P* = 0.65461 for Chao1 richness) (Extended Data Fig. 5c). Phyla analysis highlighted significant changes between ketogenic/vegan diet compared with baseline, but only a few differences when comparing ketogenic versus vegan diet (Fig. 4d). Most of the differences driving the change in beta dispersion between ketogenic and vegan/baseline diets resulted from shifts in abundance of species within the same phyla. Changes in species abundance between ketogenic and vegan diets were predominantly observed for Actinobacteria, Bacteroidetes, Firmicutes and Proteobacteria, with Firmicutes being the most impacted phylum (26 species changed, of which 18 are more abundant in vegan diet) (Fig. 4e). In line with previous studies^15,37–39^, we also observed changes following ketogenic diet in the abundance of species known to be enriched in ketogenic or animal-rich diets (for example, *Bacteroides sartorii*, *Bacteroides vulgatus*^23^), and changes following vegan diet in abundance of species previously reported to be enriched in fiber- or plant-rich diets (for example, *Bifidobacterium longum*, *Bifidobacterium pseudocatenulatum*^40^) (Fig. 4e).

We next mapped all reads to Enzyme Commission (EC) numbers to gain functional insights. In agreement with each diet consumed, most microbial enzymes upregulated following vegan diet were associated with digestion of polysaccharides unique to plants, whereas microbial enzymes upregulated following ketogenic diet related to digestion of polysaccharides coming from both plant and animal (Extended Data Fig. 5e). Interestingly, a ketogenic diet resulted in substantial downregulation of microbial gene abundance compared with baseline and vegan diets, which was reflected by the downregulation of numerous pathways following ketogenic diet (Fig. 4f,g, left). For instance, the biosynthesis of amino acids (12) and vitamins (9) was downregulated following ketogenic diet. This included biosynthesis of essential and branched-chain amino acids (BCAAs), as well as biosynthesis pathways for vitamins B1, B5 and B12 (Fig. 4g, right). Reduction in amino acid metabolism within the microbiome following ketogenic diet may result from higher abundance of amino acids in the ketogenic diet, making the host less reliant on microbiome-derived amino acids. Further exploration of functional trade-offs associated with each diet would be an important line of research.

To identify potential drivers of functional changes, we next identified the top genera producing all enzymes evaluated in the dataset, as well as all enzymes that were part of significantly impacted pathways following dietary intervention (Fig. 4h). We found that only six genera (*Bacteroides*, *Blautia*, *Eubacterium*, *Faecalibacterium*, *Lachnospira* and *Ruminococcus*) were predicted to express enzymes from pathways significantly changed (Fig. 4h). Further exploration of drivers of functional change in the microbiome may open the door to precision microbiome modeling using dietary interventions.

Thus, a ketogenic diet has a more significant impact on microbiome composition and predicted function than a vegan diet, with several of the downregulated pathways associated with amino acid and vitamin metabolism.

## Diets impact host amino acid metabolism and lipids

**Fig. 5:**
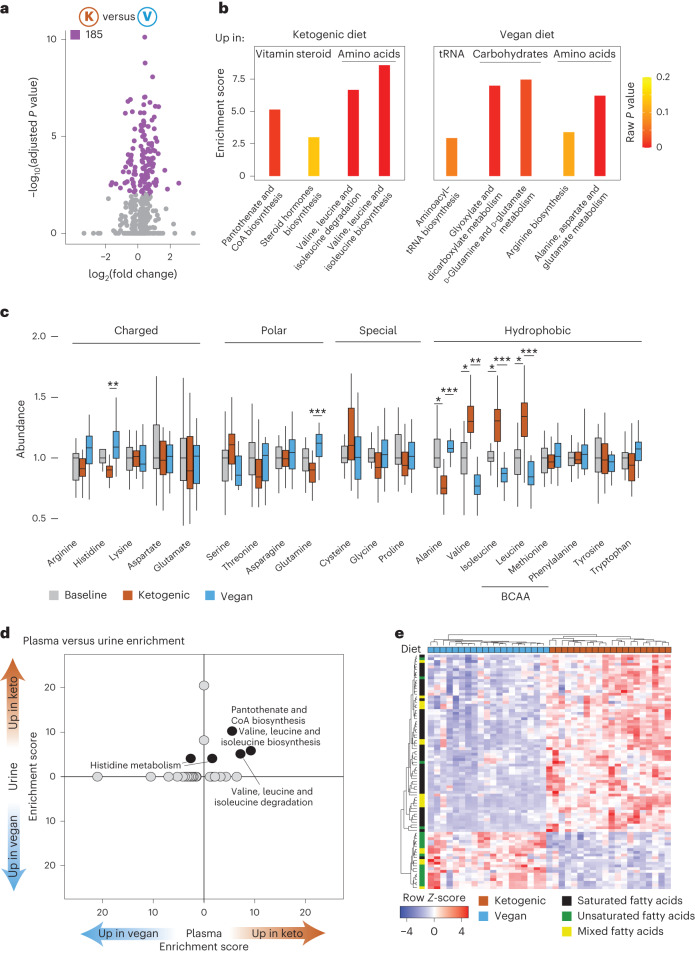
Diets significantly affect host amino acid metabolism. **a**, Volcano plot for all metabolites between ketogenic and vegan diets. Metabolites that are significantly different (FDR < 0.01) are colored purple. Significance was calculated by paired two-sided *t*-test and multiple-testing corrected. **b**, Bar graph showing all significantly differently enriched MetaboAnalyst pathway results upregulated following ketogenic diet (left) and following vegan diet (right). **c**, Abundance of all amino acids in ketogenic and vegan diets. The lower and upper hinges of the box correspond to the first and third quartiles (the 25th and 75th percentiles); the line in the box indicates the median. The upper whisker extends from the hinge to the largest value no further than 1.5 × IQR from the hinge and the lower whisker extends from the hinge to the smallest value at most 1.5 × IQR from the hinge (*n* = 20). Significance was calculated by paired two-sided *t*-test and multiple-testing corrected. **d**, Comparison of pathway enrichment between plasma (*x* axis) and 24-h urine (*y* axis) samples. Only pathways enriched in both samples are labeled. **e**, Heat map showing quantity of all significantly differentially abundant lipids per participant (column), with color legend depicting if lipids contain saturated or unsaturated fatty acids. *P* values: *P < 0.05; **P < 0.01; ***P < 0.001.

**Extended Data Fig. 6:**
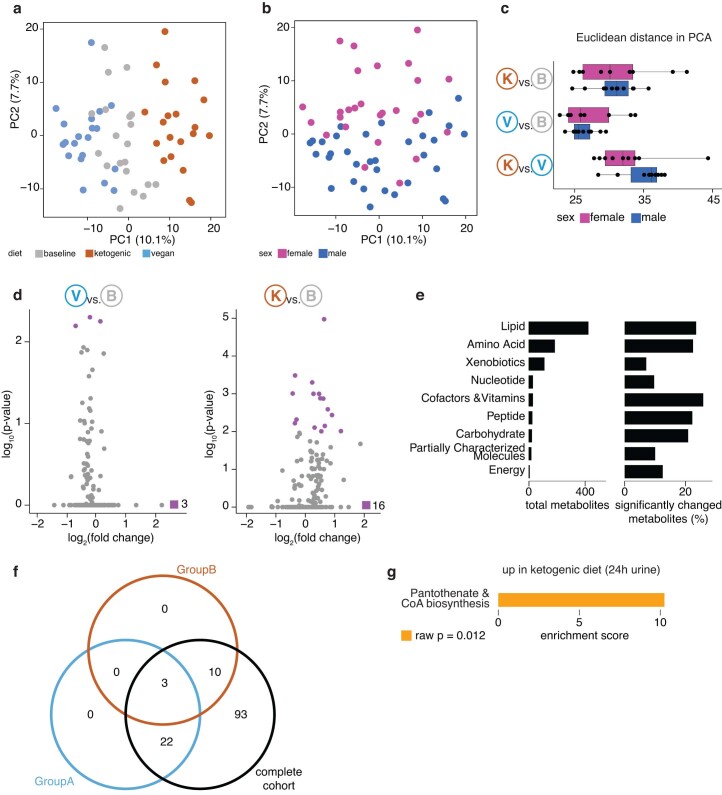
Supporting data for metabolomics data. (**a**, **b**) Principal component analysis (PCA) for plasma metabolomics data showing PC1 (explaining 10.1% of variation) and PC2 (explaining 7.7% of variation) colored by diet (**a**) and sex (**b**). (**c**) Box-whisker plot showing Euclidean distance from PCA separated by sex. The lower and upper hinges of the box correspond to the first and third quartiles (the 25^th^ and 75^th^ percentiles), the line in the box indicate the median. The upper whisker extends from the hinge to the largest value no further than 1.5 × interquartile range (IQR) from the hinge and the lower whisker extends from the hinge to the smallest value at most 1.5 × IQR of the hinge (n = 20 – 11 males/9 females). (**d**) Volcano plot for metabolites comparing vegan diet versus baseline diet (left) and ketogenic diet versus baseline diet (right). Significance was calculated with paired two-sided t-test with multiple testing correction. (**e**) Bar graph showing number of total metabolites per category (left) and percentage of significantly changed metabolites per category (right). (**f**) Venn diagram showing overlap of differentially abundant metabolites for participants for group A (start diet: vegan), group B (start diet: ketogenic) and complete data set. (**g**) MetaboAnalyst analysis for 24 h urine samples.

Metabolomic analysis can provide valuable insights into how diet shapes host metabolism. We next performed targeted metabolomics analysis in plasma and urine for all participants (Supplementary Table 1). In contrast to our proteomic and microbiome datasets (Figs. 3d and 4a), PCA generated from plasma metabolomic data separated all participants by diet, with baseline metabolomics profiles clustered directly between ketogenic and vegan diet profiles (Extended Data Fig. 6a), and only minor sex-specific effects (Extended Data Fig. 6b,c). We did not find any significant differences in metabolite profiles between diet groups (*P* = 0.4892). In total, 185 (of 859) metabolites were significantly changed between vegan and ketogenic diets in the plasma (54 metabolites upregulated in vegan, 131 upregulated in ketogenic diet) (Fig. 5a), with lipids being the most impacted (Extended Data Fig. 6e). We only observed three significantly changed metabolites between vegan and baseline diets and 16 significantly changed metabolites between ketogenic and baseline diets (of 676 total) (Extended Data Fig. 6d). ANOVA confirmed that diet order does not impact the effect of diet on metabolite profiles (Extended Data Fig. 6f). Thus, metabolomics—a more direct read-out of the impact of diet on the host than transcriptomic or proteomic data—might be a better dataset to understand mechanistic regulation of the host response to dietary interventions.

To gain functional insights into nutrition–metabolite–host physiology crosstalk, we performed functional enrichment analysis from plasma samples (Fig. 5b). Both ketogenic and vegan diets were associated with significantly upregulated amino acid biosynthesis pathways (Fig. 5b). Specifically, a ketogenic diet significantly upregulated pathways associated with biosynthesis and degradation of valine, leucine and isoleucine (BCAAs) (Fig. 5b) — aligned with the higher abundance of BCAAs in the plasma of participants consuming a ketogenic diet (Fig. 5c and ref. ^25^). Thus, abundance of amino acids in diet might result in upregulation of amino acid metabolism in the host and a paradoxical downregulation of amino acid metabolism by the microbiota (Fig. 4g). In contrast, we observed that alanine, aspartate and glutamate metabolism, as well as arginine biosynthesis, were specifically upregulated following vegan diet (Fig. 5b).

Contrary to what was observed using plasma samples, differences between diets were less evident when assessing metabolites from urine samples, with significant upregulation of only the Pantothenate and CoA biosynthesis pathway following ketogenic diet compared with vegan diet (Extended Data Fig. 6g). Overlapping all enriched pathways (independent of significance) revealed four that were concurrently enriched in plasma and urine samples, all of which were associated with the upregulation of amino acid and vitamin biosynthesis following ketogenic diet (Fig. 5d).

In line with higher intake of fatty acids during ketogenic diet, a high number of lipids were also enriched in ketogenic diet versus vegan diet (81 in ketogenic versus 22 in vegan diet) (Extended Data Fig. 1h). While both saturated and unsaturated fatty acid contents were significantly higher in ketogenic diet (Extended Data Fig. 1h), only lipids containing saturated fatty acids were enriched in plasma of participants during the ketogenic diet (Fig. 5e). In contrast, the vegan diet significantly upregulated lipids containing unsaturated fatty acids (Fig. 5e). Whether these divergences account for the differential impact on host immunity would be of interest to explore in future studies.

**Fig. 6:**
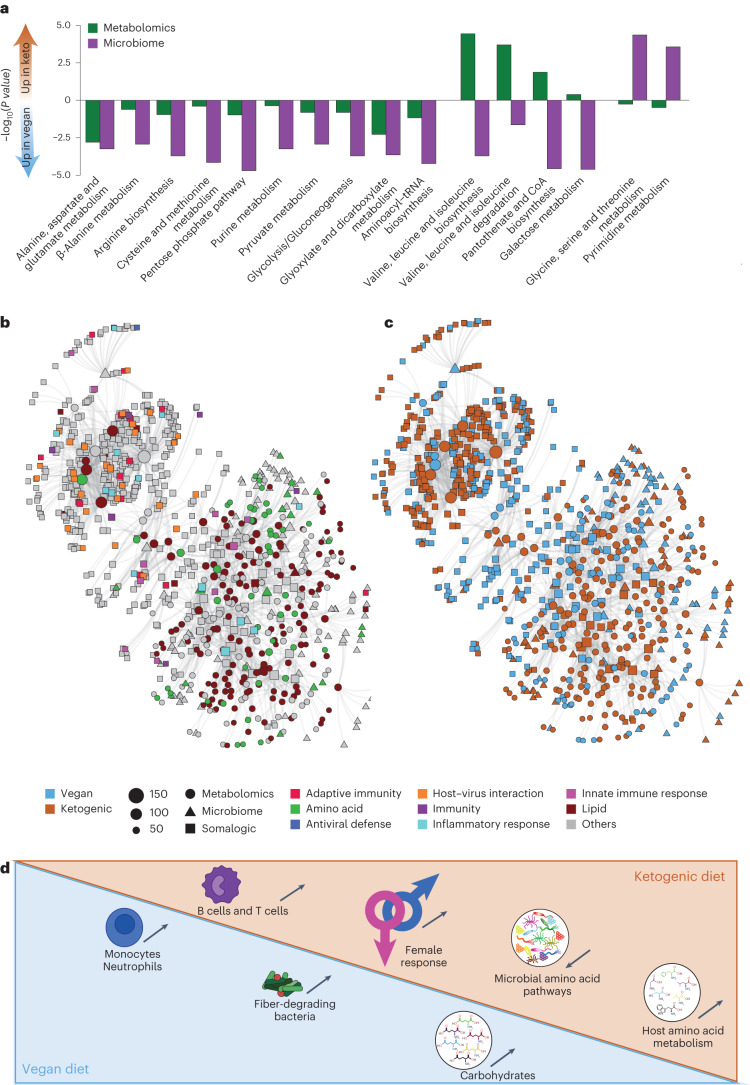
Highly interconnected network between data is driven by immunity, amino acids and lipids. **a**, Comparison of pathways significantly differently enriched between ketogenic diet and vegan diet for metabolomics (green) and microbiome data (purple). **b**,**c**, Interconnected network from all microbial enzymes, metabolites and proteins with more than ten connections colored by immune category (**b**) and diet (**c**). **d**, Graphical summary of main findings.

Overall, and in line with our microbiome data, we observed a stronger impact of a ketogenic diet on plasma host metabolite profiles than with a vegan diet. Most enriched pathways were associated with amino acid metabolism, with a total of 16 pathways enriched in both microbiome and metabolomics datasets (Fig. 6a). Strikingly, and despite the diversity and small number of participants, ten of those were convergently enriched following a vegan diet (Fig. 6a).

**Extended Data Fig. 7:**
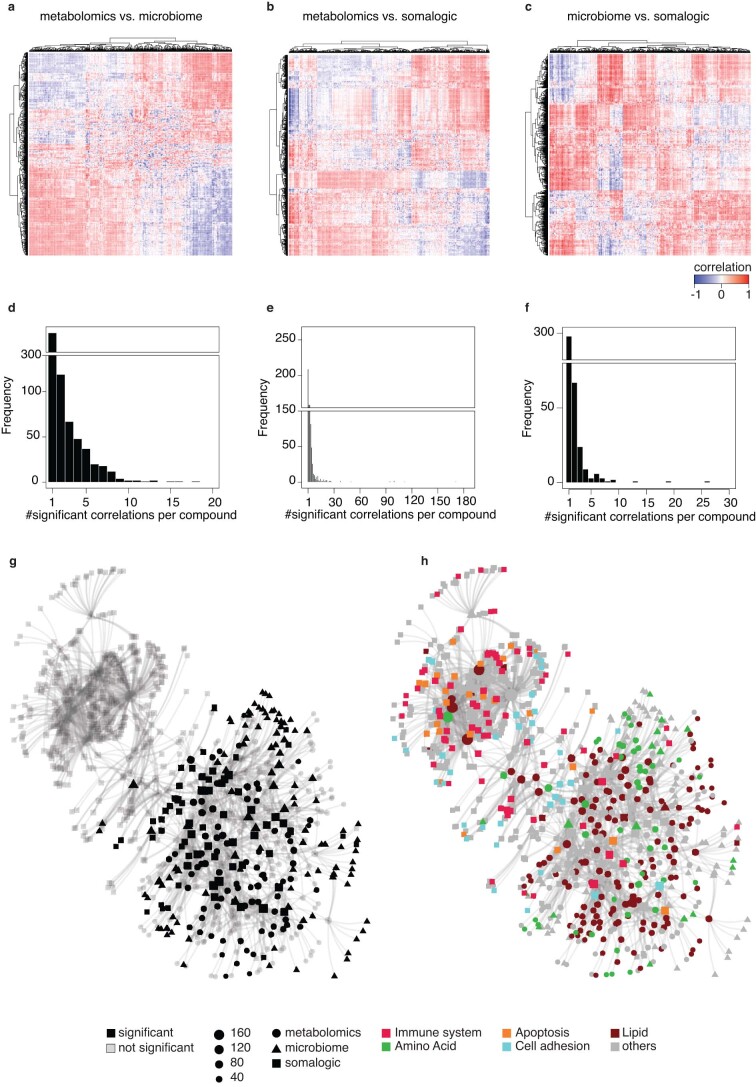
Supporting data for network analysis. (**a–c**) Correlation heat maps showing correlations of all metabolites and microbial enzymes (**a**), metabolites and proteins (**b**), and microbial enzymes and proteins (**c**). (**d–f**) Histogram of number of significant correlations for all metabolites and microbial enzymes (**d**), metabolites and proteins (**e**), and microbial enzymes and proteins (**f**). (**g, h**) Network of highly connected data points colored by significance (**g**) and categories (**h**).

We next focused specifically on correlations between enzymes, metabolites and proteins (Extended Data Fig. 7a–c). All pairwise correlation matrices showed areas of high correlation. We observed a substantial amount of negatively correlated metabolites and microbial enzymes (Extended Data Fig. 7a), whereas metabolites and proteins, as well as microbial enzymes and proteins, were mainly positively correlated (Extended Data Fig. 7b,c).

We identified data points that where highly interconnected (Extended Data Fig. 7d–f) and generated a network considering only data points with more than ten significant correlations (Fig. 6b). We observed a densely connected network with linkages between all datasets, with an upper region consisting of only data points that are similarly abundant and a lower region consisting of many data points that are significantly differently abundant between ketogenic and vegan diets (Extended Data Fig. 7g). Microbial enzymes, metabolites and proteins in the network were associated with a vast variety of biological functions, including immune system, amino acids, lipids, apoptosis and cell adhesion (Extended Data Fig. 7h). We next focused on the most represented functional terms (lipids, immune system and amino acids) (Fig. 6b). The lower region of the network was mainly driven by compounds associated with lipids or amino acids. The upper part of the network showed a dense connection of mostly immune-related data points, which were predominantly associated with adaptive immunity and host–virus interactions. In line with our conclusion from transcriptomic and proteomic datasets, most of the data points associated with adaptive immunity were more abundant following ketogenic diet compared with vegan diet (Fig. 6c).

Thus, despite the heterogeneity and small number of participants, our complex dataset allowed us to uncover a highly interconnected network between proteins, metabolites and microbial enzymes, which was mainly driven by amino acids, lipids and immune-related factors.

## Discussion

Uncovering the principles by which nutrition regulates immunity in humans could greatly improve our ability to design personalized nutritional interventions that prevent and treat disease. Here we present the first study, to our knowledge, exploring the impact of a highly controlled, cross-over, dietary intervention on human immunity, metabolism and microbiome. Of particular importance is the observation that despite the diversity of participants, our complex dataset quantifying proteins, microbial enzymes and metabolites revealed highly convergent and interconnected pathways. However, this study included only a small number of participants. At this stage it is unclear how these results generalize to a boarder population. Bigger studies are necessary to address this question sufficiently. Collectively, our work revealed a broader impact of ketogenic diet on proteome, metabolome and microbiome data, whereas both diets had a significant impact on host immunity (Fig. 6d). Why the ketogenic diet led to more widespread changes in host immunity, metabolism and microbiome than vegan diet remains unclear at this stage. One possibility is that the ketogenic diet resulted in increased utilization of fat and ketones as its primary energy source and less carbohydrate, which was the main fuel during both baseline and vegan diets^25^.

Our study revealed that a 2-week dietary intervention can impose a striking shift in host immunity, superseding genetics, age, sex, ethnicity, race and even body mass index. Of note, this study did not contain a wash-out period between diets. Interestingly, the order in which the diets were consumed did not affect our results, showing that 2 weeks of diet is sufficient to rewire host immunity, the microbiome, as well as host proteomic and metabolomic profiles. Nevertheless, the time span required to impact the host as well as the duration of the impact should be investigated further. While our findings highlight the important role of diet in rapidly rewiring host immunity, one major limitation of the present study is that exploration of immune signature was limited to blood. Whether the impact of a ketogenic or vegan diet observed in peripheral blood reflects changes in tissue immunity, and whether all tissues respond in a convergent manner to each diet, remains to be addressed. Nonetheless, our work uncovers that, at least in the blood compartment, a ketogenic diet heightened signatures linked to adaptive immunity. These findings are aligned with the previously reported role of ketogenic diet in increasing γδ T cell responses in mice^41,42^. On the other hand, we observed a previously unreported upregulation of innate immunity following vegan diet. Our findings also highlight a significant upregulation of erythropoiesis and heme metabolism following vegan diet. Heme is important in regulating transcription and protein synthesis during erythropoiesis^43^, and, in addition to oxygen transportation, erythrocytes are important modulators of innate immunity^44^. In line with this observation, the overall intake of dietary iron, another important component of erythropoiesis, was significantly higher in vegan diet than in ketogenic diet. Dietary iron comes in two different forms, heme iron (mainly found in meat and animal products) and nonheme iron (found in plant and animal products)^45^. They differ in their bioavailability and absorption rate. About 30% of heme-bound iron is absorbed, whereas only 1–10% of nonheme-bound iron is absorbed. Whether and how different sources of iron have different impacts on the immune system and host metabolism is currently unclear, but would be an important variable to investigate in future studies.

An important variable to consider when assessing the impact of diet on host immunity is the relative caloric intake. A previous study using the same cohort demonstrated that ad libitum consumption of a vegan diet was associated with a significant reduction of caloric intake when compared with a ketogenic diet^25^. Previous work from our laboratory and others showed that caloric reduction was associated with significant changes in host immunity^46–48^ and, in particular, increase in monocyte function in humans^47^. Thus, whether increased innate immunity signature following vegan diet resulted from a qualitative versus a quantitative (or both) difference in nutrition remains unclear at this point and would require further investigation.

Diet is the most important regulator of the host microbiome and, aligned with this, we found that ketogenic diet had a pronounced effect on the composition and function of the microbiome. Previous work in humans^37^ showed changes in microbiome composition, which were reproduced in this study, including an increase of bile-tolerant bacteria during ketogenic diet, as well as a decrease in Firmicutes. We did not observe any major differences between baseline and vegan diets, despite a large increase in fiber intake during the vegan diet. However, it is important to highlight that sampling of the participants was limited to fecal material and that changes in microbial communities and/or enzymatic function may be enriched at sites not highly represented in our samples, such as those linked to the epithelium or the small intestine lamina propria. To get a deeper understanding of the impact of diet on the microbiome, a more comprehensive sampling of the microbiome would be needed. Nonetheless, our findings reveal that most microbial enzymes were downregulated following ketogenic diet, leading to significant downregulation of pathways associated with amino acid metabolism and biosynthesis. In contrast, metabolomics data reveal that ketogenic diet had a strong impact on the metabolomic profile in the plasma of all participants, with an upregulation of BCAA and other amino acid pathways. Since ketogenic diet is enriched in amino acids, this observation highlights the tradeoff of function between the microbiota and its host. Of note, the pathways for alanine, aspartate and glutamine metabolism were upregulated following vegan diet, both in the microbiome and in metabolomics datasets (Fig. 6a). Further research would be necessary to understand the exact regulation and tradeoff in amino acid metabolism in both the host and the microbiome.

Nutrition profoundly impacts all aspects of our physiology. Therefore, there is great urgency to continue building a rigorous understanding of the impact of diet on human immunity and inflammation. Although highly preliminary at this stage, our findings indicate differences in activation of pathways associated with cancer following vegan and ketogenic diets. To date, there are no studies investigating the impact of vegan diet on cancer or other diseases. However, previous case studies proposed potential anti-cancer properties of a ketogenic diet (reviewed in^34^). Thus, much remains to be done to understand the mechanisms of action and the possible relevance of consuming defined diets to specific disease states. We believe that our present findings further highlight the great potential of highly controlled dietary interventions to better understand integrative physiology, improve human health and mitigate disease.

## Methods

### Recruitment and selection of participants

The study protocol was approved by the Institutional Review Board of the National Institute of Diabetes and Digestive and Kidney Diseases (NCT03878108) and is available on the Open Science Framework website (https://osf.io/fjykq/). Participants were fully informed of the risks of the study and signed consent forms before any study procedures. The study was conducted from April of 2019 to March of 2020 in the Metabolic Clinical Research Unit of the NIH Clinical Center. The first primary outcome of this study compared the mean energy intake between each 2-week diet period. The second primary outcome compared the mean energy intake on the second week of each diet period. Results for both primary outcomes were previously reported^25^. The primary exploratory aim of this study (which is reported in this manuscript) was to compare changes in immunity, microbiome composition and function, and metabolite profiles between each 2-week period of diet. Details about the study participants, inclusion and exclusion criteria, as well as experimental setup were previously published^25^. In short, male and female participants aged 18–50 yr, with stable weight and no metabolic, cardiovascular or any other disease that may influence metabolism (for example, cancer, diabetes, thyroid disease), were eligible for this study. Study participants were admitted to the Metabolic Clinical Research Unit at the NIH Clinical Center where they resided in individual rooms. Each participant was randomly assigned to receive either the ketogenic or vegan diet for the first 14 d, immediately followed by the alternative diet for another 14 d. Sex was determined by self-reporting. For proteomics and metabolomics data, samples from 20 participants were collected (11 male/9 female) and data were analyzed for difference in responses by sex. For microbiome data, samples from ten participants were analyzed (5 male/5 female). For RNA-seq data, samples from six participants (3 male/3 female) were analyzed, and for flow cytometry data samples from seven participants (3 male/4 female) were analyzed. Due to the small sample size for those datasets, no sex differences were analyzed. Informed consent was obtained from all participants; however, not all participants consented to broad data sharing. Therefore, flow cytometry, proteomics, metabolomics data and nutritional information, as well as metadata, will be shared only by request, whereas RNA-seq and microbiome data are publicly available as all participants in those datasets consented to broad data sharing.

### Statistics and reproducibility

This study was sufficiently powered to assess the effects of primary and secondary outcomes. A detailed power calculation is available in ref. ^25^. The analysis presented in this manuscript was exploratory and no statistical method was used to predetermine sample size, but the effects observed were large and highly statistically significant. Two microbiome samples were excluded from the analysis due to their collection dates (sample for baseline was taken after more than 8 d on diet). Due to the nature of this study and the obvious difference in food presented to the participants, the investigators were not blinded to allocation during experiments and outcome assessment.

### Dietary intervention

All meals and snacks for the diets were designed and analyzed using ProNutra software (v.3.4, Viocare), with nutrient values derived from the USDA National Nutrient Database for Standard Reference, Release 26 (https://www.ars.usda.gov/ARSUSERFILES/80400535/DATA/SR26/SR26_DOC.PDF) and the USDA Food and Nutrient Database for Dietary Studies, 4.0 (https://www.ars.usda.gov/northeast-area/beltsville-md-bhnrc/beltsville-human-nutrition-research-center/food-surveys-research-group/docs/fndds-download-databases/). Foods and beverages were categorized according to the NOVA system and glycemic index was calculated relative to 50 g of oral glucose. Both diets had a common foundation of nonstarchy vegetables with low amounts of digestible carbohydrates. For the ketogenic diet, animal-based products including meat, poultry, fish, eggs, dairy and nuts were added, whereas for the vegan diet legumes, rice, root vegetables, soy products, corn, lentils, peas, whole grains, bread and fruit were added.

Bottled water and snacks representative of the prevailing diet were provided ad libitum throughout the day in snack boxes located in the inpatient rooms. Meals were presented to the participants with instructions to eat as much or as little as desired.

Remaining food and beverages from each meal were identified and weighed by nutrition staff to calculate the amount of each food consumed, and the nutrient and energy intakes were calculated using the nutrition software described above. This was completed for all 1,680 meals, as well as for the daily snacks and bottled water. Two participants had errors in their food weights while on the vegan diet and, therefore, the intake data for the days with these errors (3 d total) were removed from the final dataset.

### Blood sample collection

Blood samples were collected at different time points per individual (Supplementary Table 1).

### Processing of blood samples for flow cytometry and transcriptomic analysis

Blood obtained from seven subjects at three time points (21 samples) was used to isolate PBMCs for flow cytometry. Further blood from six subjects at three time points (18 samples) was obtained for transcriptomic analysis. PBMCs were isolated from 5 ml of whole blood using LeucoSep tubes (Greiner Bio-one) and Ficoll-Paque Plus (GE Healthcare) for density gradient centrifugation, before cryopreservation in 90% heat-inactivated FBS (Gibco) with 10% dimethyl sulfoxide (Sigma-Aldrich), according to a standard protocol (https://chi.niaid.nih.gov/web/new/our-research/SOP-Isolation.pdf). For RNA analysis, 100 μl of whole blood was lysed in Trizol-LS (Qiagen) according to manufacturer’s instructions and immediately kept at −80 °C.

### Flow cytometry of PBMCs

PBMCs from 21 samples were thawed and washed in RPMI containing 50 U ml^−1^ benzonase nuclease then PBS. Cells were incubated with LIVE/DEAD Fixable Blue Dye (Life Technologies), washed and re-suspended in 100 μl of FACS buffer (PBS with 0.5% fetal calf serum, 0.5% normal mouse serum and 0.02% NaN_3_), before incubation for 30 min with fluorochrome-conjugated antibodies to CD3, CD4, CD8, CD11c, CD14, CD16, CD19, CD20, CD24, CD25, CD27, CD38, CD45, CD45RA, CD45RO, CD56, CD123, CD127, CCR4, CCR6, CCR7, CCR10, CXCR3, CXCR5, HLA-DR and IgD (Extended Data Table 2). Cells were washed an additional two times with FACS buffer, fixed in 1% paraformaldehyde and acquired using an Aurora spectral cytometer (Cytek Biosciences).

### Analysis of flow cytometry data

After data acquisition, the frequency of major populations was analyzed with FlowJo software v.10 (BD Biosciences) based on previously described manual gating strategies^26^ (Extended Data Table 1 and Extended Data Fig. 2). For statistical analysis, fold change between frequencies per population per individual was calculated and assessed using a two-sided paired *t*-test with Bonferroni–Hochberg multiple-testing correction.

### LME model to estimate impact of diet order on data

To estimate if there were any biases introduced by the order of diets, we applied an LME model (~diet_group + (~diet_group | diet)) with the lmerTest R package^49^ for proteomics, metabolomics and microbiome data.

### RNA-seq library preparation of whole blood

RNA was extracted from 18 samples stored in Trizol-LS using the miRNeasy Micro Kit (Qiagen), following the manufacturer’s protocol for samples containing <1 μg of RNA. Briefly, buffer RWT was prepared with isopropanol, and after binding to RNeasy columns the RNA was treated with DNaseI (Qiagen) before washing and eluting in 20 μl of nuclease-free water. RNA concentration was determined using Qubit RNA High Sensitivity assay (Thermo Fisher), and quality was assessed using Agilent 4200 TapeStation (Agilent Technologies). RNA-seq libraries were prepared from 100 ng of total RNA using Universal Plus mRNA-seq Kit with NuQuant Human Globin AnyDeplete kit (Tecan Genomics). First, messenger RNA transcripts were captured, fragmented and converted to complementary DNA. Following second-strand synthesis and end repair, DNA fragments were ligated with dual-indexed adapters compatible with the Illumina platform. Libraries were subjected to strand selection and removal of ribosomal RNA and globin transcripts, followed by 16 cycles of amplification. Purified libraries were analyzed with the Qubit and Agilent TapeStation to assess concentration and size distribution, respectively, then normalized and pooled for sequencing. Final molarity of the pool was determined by quantitative polymerase chain reaction using the KAPA library quantification kit (Roche). Paired-end sequencing was performed on the NextSeq 500 (Illumina) using the High Output 150-cycles kit in 2 × 75-base pair (bp) format.

### Analysis of RNA-seq

Sequencing results were demultiplexed and converted to FASTQ format using Illumina bcl2fastq software (Illumina). The sequencing reads were adapter and quality trimmed and then aligned to the human genome (https://www.ncbi.nlm.nih.gov/datasets/genome/GCF_000001405.26/; version hg38) using the splice-aware STAR aligner^50^ and single nucleotide polymorphism (SNP) calls were generated using the previously published protocol^51^. SNP calls were used for quality control of samples and subject mapping. Differentially expressed genes were identified using the limma linear model^52^ which models the log of the counts per million (c.p.m.) of each gene. Enriched gene sets were identified using the preranked gene-set enrichment analysis (GSEA) algorithm implemented in the fgsea R package^53^. Genes were ranked using the moderated T statistics for the relevant coefficient from the limma model. Enrichment was assessed with a gene-set list that included MSIGDB’s Hallmark collection (https://www.gsea-msigdb.org/gsea/msigdb/human/collections.jsp) and BTMs^27^ (https://github.com/shuzhao-li/BTM). Analysis was performed on six different comparisons: ketogenic diet versus baseline diet; ketogenic diet versus vegan diet; ketogenic diet versus previous diet; vegan diet versus ketogenic diet; vegan diet versus baseline diet; vegan diet versus previous diet. Previous diet refers to the diet consumed directly beforehand (for example, when analyzing ketogenic diet versus previous diet, previous diet refers to vegan diet for group A and to baseline diet for group B). PCA was performed in R using the function prcomp. For data visualization of heat maps, transcripts per million (TPM) was used and either TPM values or row *Z*-scores were shown. IPA^54^ (Qiagen) was used to analyze enrichment of disease terms. To analyze contribution of sorted cell populations to the overall pathway signature, gene counts for sorted immune cell populations from the blood were downloaded from the Human Protein Atlas^35^ and visualized as heat maps showing row *Z*-scores.

### Proteomic analysis of plasma

Peripheral blood plasma obtained from 20 subjects at three time points (60 samples) was analyzed using the SomaScan HTS Assay (SomaLogic), an aptamer-based quantitative proteomic biomarker discovery platform^55^. The assay quantifies 1,306 proteins that belong to broad biological subgroups including receptors, kinases, cytokines, proteases, growth factors, protease inhibitors, hormones and structural proteins. A complete list of the analytes measured can be found in Supplementary Table 2. The assay was run according to manufacturer specifications before data were normalized for hybridization, interplate and median signal variation, and inspected using a web tool, both as previously described^56,57^.

### Analysis of proteomics data

Differentially abundant proteins were calculated using a two-sided paired *t*-test with Bonferroni–Hochberg correction. For tissue signatures, tissue specificity was downloaded from the Human Protein Atlas^58^. Proteins with enhanced or enriched tissue specificity were considered for analysis of tissue origin, for all proteins significantly upregulated in ketogenic and vegan diets separately. PCA was performed in R using the package prcomp. To assess differences in PCA between sexes, Euclidean distances between data points for each individual between baseline diet and ketogenic diet, baseline diet and vegan diet, and ketogenic diet and vegan diet were calculated, and Student’s *t*-test was applied between female and male data. Functional analysis was performed with STRING (https://string-db.org/) with data from fold change for all proteins between diets and using the STRING analysis algorithm ‘proteins with value/ranks’.

### Microbiome sample collection

Stool samples were collected at different time points per individual (Supplementary Table 3). DNA was extracted from ~50 mg of stool samples in two stages: an initial homogenization in Lysis Matrix E tubes (MP Biomedicals) with a Precellys 24 Tissue Homogenizer (Bertin Instruments) and processing of the resultant supernatant using the MagAttract PowerMicrobiome DNA/RNA EP kit (Qiagen) on an Eppendorf automated liquid handling system as per the manufacturer’s instructions.

Isolated DNA was checked for concentration and quality on a BioTek Synergy HTX plate reader.

Metagenomic libraries were prepared using the Nextera DNA Flex Library Prep Kit (Illumina) per the manufacturer’s instructions with 100 ng of DNA as sample input. The concentration of the libraries was quantified using the Qubit dsDNA HS assay on a Qubit 2.0 fluorometer (Life Technologies). Library size and quality were assessed via the Agilent High Sensitivity D5000 ScreenTape on an Agilent 4200 Tapestation.

Metagenomic libraries were normalized to an equimolar concentration and pooled. The pool was diluted to 1.8 pM, mixed with a 1% PhiX control library and paired-end sequenced (2 × 75 bp) using a NextSeq 500/550 High Output v2 150-cycle Reagent Cartridge on a NextSeq 500 sequencer (Illumina).

### Microbiome analysis

For sequence analysis, read pairs were trimmed for quality at Q15 and Illumina Nextera adapter sequences were removed using BBDuk (https://github.com/BioInfoTools/BBMap/tree/master). The pairs were subsequently assembled with the metaSPAdes pipeline from SPAdes^59^. Taxonomic assignment was done with Kraken2 (ref. ^60^) using Bracken^61^ for abundance estimation and the maxikraken2 (v_1903_140GB) database (https://lomanlab.github.io/mockcommunity/mc_databases.html). Sequences matching to the human genome were excluded from all analyses. For beta diversity and PCoA of the taxonomic abundances, Jaccard distance measure on the relative abundance was used. To assess changes in beta diversity, a permutation ANOVA (PERMANOVA)—a permutation analysis of variance—was performed using the adonis2 function from the vegan R package 2.6-2 using a marginal model ~Diet + SubjectID.

Putative gene abundances were produced using Prodigal^62^ for the computational gene finding on the assembled contigs, which was parallelized by dividing the contigs into separate FASTA files using seqkit^63^, with bowtie2 (ref. ^64^) to map the reads back to the assembly, samtools^65^ to sort the bam files, picard (http://broadinstitute.github.io/picard/) to generate mapping statistics and remove machine duplicates and VERSE^66^ to estimate gene abundances from the mapping using the htseq algorithm^67^. HUMAnN 3.0 (ref. ^68^) was used for functional annotation of the putative gene sequences using the KEGG (https://www.genome.jp/kegg/pathway.html), EC (https://enzyme.expasy.org/) and MetaCyc^69^ (https://metacyc.org/) pathways, databases and default parameters. Putative enzyme (EC) abundances were compared between diets, and those found to be significantly higher in each diet were analyzed by AMON^70^ with the ‘unique only’ option for KEGG pathway inference based on inferred compounds uniquely enriched in each diet (data shown in Fig. 6).

To identify CAZymes (carbohydrate-active enzymes) (http://www.cazy.org/), the putative genes were profiled using the dbCAN^71^ standalone program, V10 database (https://bcb.unl.edu/dbCAN/) and the HMMER, DIAMOND and eCAMI tool options. We required that at least two tools identify the CAZy^72^ domain as being present.

Statistical comparisons of taxonomic and gene abundances between diets were carried out by first transforming the abundances by centered log-ratio, and then using MaAsLin2 (ref. ^73^) with transformation and normalization set to ‘NONE’ and method ‘LM’ using the LME model ~Diet + (1 | SubjectID). Alpha diversity and ordination were computed with the vegan R package 2.6-2^74^ using the diversity and betadisper functions. Statistical analyses of the diversity were performed with vegan’s adonis2 function and the lmerTest R package^49^. All *P* values were corrected for multiple comparisons using the qvalue function from the qvalue R package.

### Metabolomics sample collection

Discovery metabolomics analyses were conducted on stored (−70 °C since collection) plasma and urine (collected over 24 h) samples from 20 participants (60 samples) by Metabolon. Samples were acquired in two different batches, with samples from ketogenic diet and vegan diet acquired together, and samples from baseline diet acquired in a separate experiment. Samples were analyzed using ultra-high-performance liquid chromatography (UPLC) with tandem mass spectrometry (MS/MS) for a broad range of metabolites (<1 kDa), representing multiple metabolic pathways including endogenously derived amino acids, carbohydrates, lipids, cofactors and vitamins, intermediates of energy metabolism, as well as xenobiotics derived from exogenous sources such as food or drugs. In brief, serum samples were prepared using the automated MicroLab STAR system via the Hamilton Company. Recovery standards were added, and the protein fraction was extracted with methanol followed by vigorous shaking and centrifugation. Sample extracts were dried and reconstituted using recovery solvents containing fixed concentrations of standards. These extracts were analyzed using reversed-phase UPLC–MS/MS in positive-ion-mode electrospray ionization (ESI) and negative-ion-mode ESI. Raw data were extracted, peak-identified and processed by Metabolon using proprietary software and a biochemical reference library of more than 4,500 known metabolites based on authentic standards.

### Metabolomics analysis

For downstream analysis of ketogenic versus vegan diet (40 samples in total), all compounds with more than four imputed values (10% of the data) were filtered out. For analysis of baseline diet versus ketogenic/vegan diet (60 samples in total), all compounds with more than six imputed values (10% of the data) were filtered out. To calculate significant differences between diets, a paired Student’s *t*-test was performed per compound and the *P* values were Bonferroni–Hochberg corrected.

For the plasma dataset analyzing ketogenic diet versus vegan diet, a total of 188 compounds were filtered out due to missing/imputed values, leaving a total of 859 compounds to be analyzed (Supplementary Table 4). For the plasma dataset analyzing baseline diet versus ketogenic/vegan diet, a total of 413 compounds were filtered out, leaving a total of 678 compounds to be analyzed (Supplementary Table 5). For pathway analysis comparing ketogenic diet versus vegan diet, compounds significantly upregulated in vegan or ketogenic diet were converted to Human Metabolome Database (HMDB) IDs and uploaded to MetaboAnalyst^75^ using the enrichment analysis for pathway-based analysis for KEGG pathways. A custom background was uploaded (Supplementary Table 4). To generate the custom background, all compounds with HMDB IDs were uploaded to MetaboAnalyst and converted to chemical names. This file then was used as a background and 102 of 683 compounds were not matched to HMDB compound names, leaving 581 compounds in the background set.

For the urine dataset comparing ketogenic versus vegan diet, a total of 412 compounds were filtered out due to missing/imputed values, leaving a total of 970 compounds to be analyzed. A custom background was uploaded of 621 compounds, of which 52 were not recognized by MetaboAnalyst (Supplementary Table 4).

For comparison of pathways between plasma and urine, all pathways predicted to be enriched by MetaboAnalyst were used without filter. Enrichment score of plasma versus urine was plotted.

For the lipid heat map, all significantly differentially abundant compounds categorized as lipids were used. Lipids were than manually assigned as saturated fatty acids, unsaturated fatty acids or mixed fatty acids.

### Correlation analysis

All datasets with data for at least ten participants were correlated with each other (proteomics, metabolomics and microbiome data). For all comparisons the log_2_ fold change between ketogenic diet and vegan diet was calculated.

To correlate microbial enzyme abundance with protein abundance, log_2_ fold changes of the c.p.m. abundances of each EC number and log_2_ fold changes of protein abundance were computed from samples collected at time 3 over time 2 for each subject.

To correlate microbial enzyme abundance with metabolite abundance, log_2_ fold changes of the c.p.m. abundances of each EC and log_2_ fold changes of metabolite abundance were computed from samples collected at time 3 over time 2 for each subject. To correlate metabolites with protein abundance, log_2_ fold changes of protein abundance and metabolite abundance were computed from samples collected at time 3 over time 2 for each subject. For all sample comparisons, correlation was calculated for each subject using Spearman’s *ρ* calculated with the pspearman^76^ R package and *P* values were FDR-corrected with p.adjust.

### Metabolomics and microbiome pathways

KEGG pathway analyses for metabolomics and microbiome (using AMON) data were compared. All pathways predicted to be enriched in the metabolomics dataset and the microbiome dataset were merged.

### Network analysis

All significant correlations were selected for downstream network analysis. All enzymes, metabolites and proteins with more than ten connections were integrated into the network. A network graph was generated and visualized with R packages igraph^77^ and tidygraph (https://tidygraph.data-imaginist.com/).

### Reporting summary

Further information on research design is available in the Nature Portfolio Reporting Summary linked to this article.

## Online content

Any methods, additional references, Nature Portfolio reporting summaries, source data, extended data, supplementary information, acknowledgements, peer review information; details of author contributions and competing interests; and statements of data and code availability are available at 10.1038/s41591-023-02761-2.

### Supplementary information

Supplementary Information Supplementary Tables 1 and 3.

Reporting Summary

Supplementary Table 2 List of all proteins measured in SomaLogic.

Supplementary Table 4 Metabolomics data for plasma and urine with mean values per diet, fold change and adj. *P* value for ketogenic and vegan diets.

Supplementary Table 5 Metabolomics data for plasma with mean values per diet, fold change and adj. *P* value for ketogenic/vegan diet versus baseline diet.

## Figures and Tables

**Extended Data Table 1:**
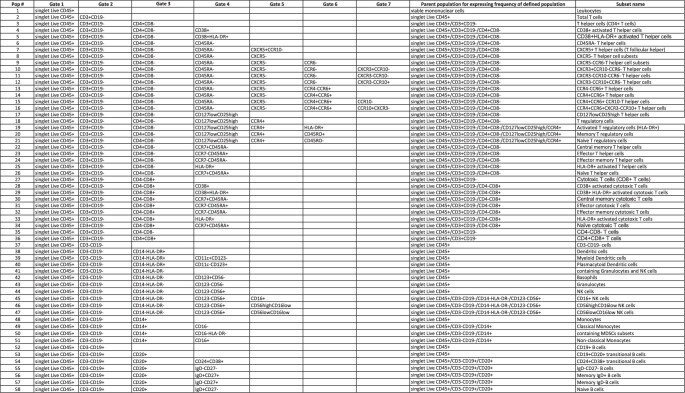
Gating strategy for high dimensional flow cytometry

**Extended Data Table 2:**
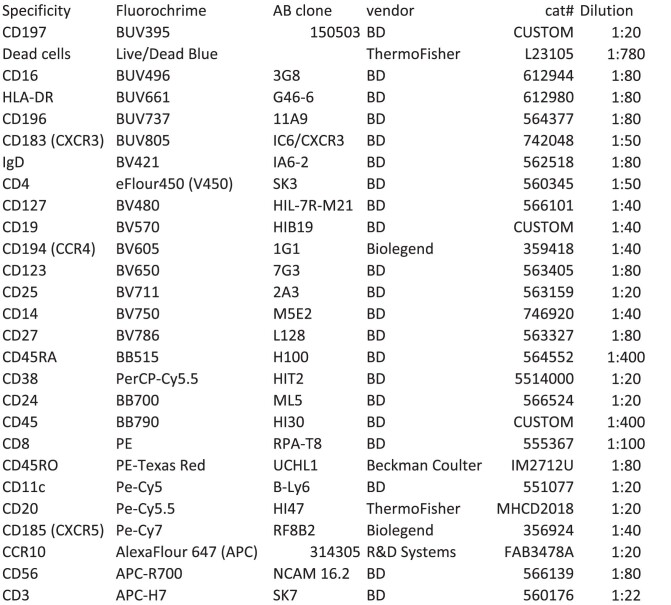
Antibodies and dilutions used for high dimensional flow cytometry

## Data Availability

All RNA-seq raw data are publicly available through dbGAP: https://www.ncbi.nlm.nih.gov/projects/gapprev/gap/cgi-bin/study.cgi?study_id=phs003187.v1.p1. Microbiome sequencing data are available through BioProject accession PRJNA981159. All other datasets (de-identified metadata, nutritional information, flow cytometry dataset, proteomics dataset and metabolomics dataset) are available upon request due to some participants not consenting to broad data sharing. Requests should be sent to the corresponding authors, Yasmine Belkaid (ybelkaid@niaid.nih.gov), Kevin Hall (kevinh@niddk.nih.gov) or Verena Link (verena.link@nih.gov), and will be fulfilled within 2 weeks. For analysis of nutritional data, USDA National Nutrient Database for Standard Reference, Release 26 (https://www.ars.usda.gov/ARSUSERFILES/80400535/DATA/SR26/SR26_DOC.PDF) and the USDA Food and Nutrient Database for Dietary Studies, 4.0 (https://www.ars.usda.gov/northeast-area/beltsville-md-bhnrc/beltsville-human-nutrition-research-center/food-surveys-research-group/docs/fndds-download-databases/) were used. For RNA-seq analysis, reads were mapped to the human genome (version hg38) (https://www.ncbi.nlm.nih.gov/datasets/genome/GCF_000001405.26/). For analysis of gene expression from sorted cell populations from blood, as well as to analyze tissue origin from proteins, the Human Protein Atlas (https://www.proteinatlas.org/) was utilized. For functional annotation analysis, we utilized the MSIGDB’s Hallmark collection (https://www.gsea-msigdb.org/gsea/msigdb/human/collections.jsp) and blood transcription modules database (https://github.com/shuzhao-li/BTM). For microbiome analysis, the maxikraken2 DB (https://lomanlab.github.io/mockcommunity/mc_databases.html) (v_1903_140GB) was utilized, as well as the KEGG DB (https://www.genome.jp/kegg/pathway.html), the enzyme nomenclature (EC) DB (https://enzyme.expasy.org/), the MetaCyc DB (https://metacyc.org/), the dbCAN DB (https://bcb.unl.edu/dbCAN/) and the CAZy DB (http://www.cazy.org/).

## Code availability

All custom code utilized for the analysis of these data is available upon request as not all datasets are publicly available. Requests should be sent to the corresponding authors, Yasmine Belkaid (ybelkaid@niaid.nih.gov), Kevin Hall (kevinh@niddk.nih.gov) or Verena Link (verena.link@nih.gov), and will be fulfilled within 2 weeks.
