# Author Correction: Differential peripheral immune signatures elicited by vegan versus ketogenic diets in humans

**DOI:** 10.1038/s41591-024-02884-0

**Published:** 2024-02-27

**Authors:** Verena M. Link, Poorani Subramanian, Foo Cheung, Kyu Lee Han, Apollo Stacy, Liang Chi, Brian A. Sellers, Galina Koroleva, Amber B. Courville, Shreni Mistry, Andrew Burns, Richard Apps, Kevin D. Hall, Yasmine Belkaid

**Affiliations:** 1 Metaorganism Immunity Section, Laboratory of Host Immunity and Microbiome, National Institute of Allergy and Infectious Diseases, National Institutes of Health, Bethesda, MD USA; 2 NIH Center for Human Immunology, National Institutes of Health, Bethesda, MD USA; 3 Bioinformatics and Computational Biosciences Branch, Office of Cyber Infrastructure and Computational Biology, National Institute of Allergy and Infectious Diseases, National Institutes of Health, Bethesda, MD USA; 4 Center for Cellular Engineering, Department of Transfusion Medicine, Clinical Center, National Institutes of Health, Bethesda, MD USA; 5 Department of Cardiovascular and Metabolic Sciences, Lerner Research Institute, Cleveland Clinic, Cleveland, OH USA; 6 National Institute of Diabetes and Digestive and Kidney Diseases, National Institutes of Health, Bethesda, MD USA; 7 NIAID Microbiome Program, National Institute of Allergy and Infectious Diseases, National Institutes of Health, Bethesda, MD USA

**Keywords:** Feeding behaviour, Applied immunology, Metabolism, Data integration

Correction to: *Nature Medicine* 10.1038/s41591-023-02761-2, published online 30 January 2024.

In the version of the article initially published, in the “Dietary intervention alters lymphoid composition” section, the sentence “The vegan diet was high in dietary fiber and dietary sugars as compared with the ketogenic diet” previously read “…and low in dietary sugars”. Additionally, in Extended Data Fig. 1e, the order and color of the bars representing sugar were switched. These errors have now been corrected in the HTML and PDF versions of the article.

