# Supplementary material for: Differential peripheral immune signatures elicited by vegan versus ketogenic diets in humans
Source: Nat Med. 2024 Jan 30;30(2):560–72. doi: 10.1038/s41591-023-02761-2 (PMC10878979; doi:10.1038/s41591-023-02761-2)
Supplement: Supplementary file 1 — Supplementary Tables 1 and 3. [file 41591_2023_2761_MOESM1_ESM.pdf]

nature medicine

Article https://doi.org/10.1038/s41591-023-02761-2

Differential peripheral immune signatures elicited by vegan versus ketogenic diets in humans

In the format provided by the authors and unedited

Supplementary Table 1

Participant	Collection day baseline	Collection day Diet1	Collection day Diet2
15	1	14	29
16	1	14	29
17	1	14	29
20	1	15	29
21	1	15	29
22	1	13	29
23	0	15	29

Supplementary Table 3

Participant	Collection day baseline	Collection day Diet1	Collection day Diet2
9	7	14	21
10	8	14	25
13	6	14	26
14	7	14	26
15	8	12	27
17	0	14	27
20	1	14	27
21	2	15	27
22	1	8	27
23	2	7	25
