# Supplementary material for: Differential peripheral immune signatures elicited by vegan versus ketogenic diets in humans
Source: Nat Med. 2024 Jan 30;30(2):560–72. doi: 10.1038/s41591-023-02761-2 (PMC10878979; doi:10.1038/s41591-023-02761-2)
Supplement: Supplementary file 2 — Reporting Summary [file 41591_2023_2761_MOESM2_ESM.pdf]

nature portfolio

Corresponding author(s): Yasmine Belkaid, Kevin D. Hall, Verena M. Link

Last updated by author(s): Nov 10, 2023

Reporting Summary

Nature Portfolio wishes to improve the reproducibility of the work that we publish. This form provides structure for consistency and transparency in reporting. For further information on Nature Portfolio policies, see our Editorial Policies and the Editorial Policy Checklist.

Statistics

For all statistical analyses, confirm that the following items are present in the figure legend, table legend, main text, or Methods section.

n/a	Confirmed	
☐	☒	The exact sample size (*n*) for each experimental group/condition, given as a discrete number and unit of measurement
☐	☒	A statement on whether measurements were taken from distinct samples or whether the same sample was measured repeatedly
☐	☒	The statistical test(s) used AND whether they are one- or two-sided<br>*Only common tests should be described solely by name; describe more complex techniques in the Methods section.*
☐	☒	A description of all covariates tested
☐	☒	A description of any assumptions or corrections, such as tests of normality and adjustment for multiple comparisons
☐	☒	A full description of the statistical parameters including central tendency (e.g. means) or other basic estimates (e.g. regression coefficient) AND variation (e.g. standard deviation) or associated estimates of uncertainty (e.g. confidence intervals)
☐	☒	For null hypothesis testing, the test statistic (e.g. *F*, *t*, *r*) with confidence intervals, effect sizes, degrees of freedom and *P* value noted<br>*Give P values as exact values whenever suitable.*
☒	☐	For Bayesian analysis, information on the choice of priors and Markov chain Monte Carlo settings
☒	☐	For hierarchical and complex designs, identification of the appropriate level for tests and full reporting of outcomes
☒	☐	Estimates of effect sizes (e.g. Cohen's *d*, Pearson's *r*), indicating how they were calculated

Our web collection on statistics for biologists contains articles on many of the points above.

Software and code

Policy information about availability of computer code

Data collection	For data collection software of the respective machines were used: Flow Cytometry: Aurora sepctral cytometer software SpectroFlo (v 2.0.0); RNA-seq: NextSeq 500 software (Illumina), SomaLogic: SomaScan Platform, Metagenomics: NextSeq 500 software (Illumina), Metabolomics: data collection was outsourced to Metabolon Inc.
Data analysis	Nutritional data: ProNutra software v.3.4; Flow cytometry data: FlowJo software version 10 (BD Biosciences); RNA-seq data: bcl2fastq (Illumina) v2.17.1.14, STAR aligner version 2.7.9a, Ingenuity Pathway Analysis (IPA) version January 2023, R packages: LIMMA, FGSEAR, prcomp; Somalogic: STRING version May 2023, R packages: prcomp, lmerTest; Metabolomics: MetaboAnalyst version 5.0; R packages: pspearman R package 0.3.1; igraph R package 1.5.1; tidygraph R package 1.2.3; Microbiome: BBDuK 38.0.1, metaSPAdes 3.15.3, Kraken 2.1.2, Bowtie2 2.4.4, SAMtools 1.13, Picard 2.25.7, Prodigal 2.6.3_31b300a, SeqKit 2.0.0, VERSE 0.1.5, HUMANn 3.0.0, AMON 1.0.0, R 4.2.2, R packages: MaAsLin2 R package 1.12, Vegan R package 2.6-2, lmerTest R package, qvalue R package

For manuscripts utilizing custom algorithms or software that are central to the research but not yet described in published literature, software must be made available to editors and reviewers. We strongly encourage code deposition in a community repository (e.g. GitHub). See the Nature Portfolio guidelines for submitting code & software for further information.

Data

Policy information about availability of data

All manuscripts must include a data availability statement. This statement should provide the following information, where applicable:

- Accession codes, unique identifiers, or web links for publicly available datasets
- A description of any restrictions on data availability
- For clinical datasets or third party data, please ensure that the statement adheres to our policy

All RNA-seq raw data is publicly available through dbGAP: (https://www.ncbi.nlm.nih.gov/projects/gapprev/gap/cgi-bin/study.cgi?study_id=phs003187.v1.p1). Microbiome sequencing data is available through BioProject accession PRJNA981159.
For analysis of nutritional data USDA National Nutrient Database for Standard Reference, Release 26 (https://www.ars.usda.gov/ARSUSERFILES/80400535/DATA/SR26/SR26_DOC.PDF) and the USDA Food and Nutrient Database for Dietary Studies, 4.0. Foods (https://www.ars.usda.gov/northeast-area/beltsville-md-bhnrc/beltsville-human-nutrition-research-center/food-surveys-research-group/docs/fndds-download-databases/) were used. For RNA-seq analysis, reads were mapped to the human genome (version hg38) (https://www.ncbi.nlm.nih.gov/datasets/genome/GCF_000001405.26/). For analysis of gene expression from sorted cell populations from the blood, as well as to analyze tissue origin from proteins the human protein atlas (https://www.proteinatlas.org/) was utilized. For functional annotation analysis, we utilized the MSIGDB's Hallmark collection (https://www.gsea-msigdb.org/gsea/msigdb/human/collections.jsp) and blood transcription modules database (https://github.com/shuzhao-li/BTM). For microbiome analysis the maxikraken2 DB (https://lomanlab.github.io/mockcommunity/mc_databases.html) (v_1903_140GB) was utilized, as well as KEGG DB (https://www.genome.jp/kegg/pathway.html), the enzyme nomenclature (EC) DB (https://enzyme.expasy.org/), the MetaCyc DB (https://metacyc.org/), the dbCAN DB (https://bcb.unl.edu/dbCAN/), and the CAZy DB (http://www.cazy.org/).

Human research participants

Policy information about studies involving human research participants and Sex and Gender in Research.

Reporting on sex and gender	Throughout the manuscript we use the term sex, which was determined based on self-reporting. For a subsample of participants where we had sequencing data, we confirmed that self-reported sex aligned with chromosomal genotype. The study was designed to get a balanced ratio of male and female participant. All data in this study was analyzed in two groups without consideration of sex, as well as separated by sex. 16 our of 20 participants allowed for broad data sharing, the other 4 actively refused. Individual-level metadata therefore will only be shared on request.<br>Sex- and gender-based analysis in this manuscript: Flow-cytometry: no analysis due to small sample size (7); RNA-seq: no analysis due to small sample size (6); Metagenomics: no analysis due to small sample size (10); SomaLogic (20 samples): differences in response to diet between genders was analyzed and reported; Metabolomics (20 samples): differences in response to diet between genders was analyzed and reported
Population characteristics	11 male and 9 female weight stable adults aged (mean±SE) 29.9±1.4 years with BMI 27.8±1.3 kg/m2. Study participants were excluded based on several diseases and treatments during screening process. During the analysis of this study, no health and diagnosis information was used for analysis in this manuscript due to the small sample size of only 20 participants. For more details see Hall et al., Nature Medicine, PMID: 33479499.
Recruitment	Participants were recruited through the NIH Office of Patient recruitment beginning in February of 2019. No potential self-selection bias was identified. For more details see Hall et al., Nature Medicine, PMID: 33479499.
Ethics oversight	Institutional Review Board of the National Institute of Diabetes & Digestive & Kidney Diseases (NCT03878108). The study protocol is available on the Open Science Framework website (https://osf.io/fjykq/).

Note that full information on the approval of the study protocol must also be provided in the manuscript.

Field-specific reporting

Please select the one below that is the best fit for your research. If you are not sure, read the appropriate sections before making your selection.

☒ Life sciences ☐ Behavioural & social sciences ☐ Ecological, evolutionary & environmental sciences

For a reference copy of the document with all sections, see nature.com/documents/nr-reporting-summary-flat.pdf

Life sciences study design

All studies must disclose on these points even when the disclosure is negative.

Sample size	The study was powered for it's primary study goal of identifying if there is a difference in calorie intake between a high-carbohydrate, low-fat (vegan) diet and a low-carbohydrate, high-fat (ketogenic) diet. More details are provided in the original publication of this study cohort (Hall et al., Nature Medicine, PMID: 33479499). Due to sample availability, not all assays could be performed on all participants. In total we collected 7 samples for flow-cytometry assays, 6 samples for RNA-seq assays, 10 samples for metagenomics assays, (microbiome), 20 samples for metabolomics assays, and 20 samples for proteomics assays.

Data exclusions	One participant was removed from study due to a hypoglycemia episode (see Hall et al., Nature Medicine, PMID: 33479499 for more details). One metagenomics data set was removed because the data collection days for baseline and first diet were too close together confounding the analysis.
Replication	Our results have not been replicated yet.
Randomization	Randomization of diet order was conducted by the NIH Clinical Center Nutrition Department using an online randomization program (https://www.sealedenvelope.com/simple-randomiser/v1/lists). The randomization scheme was not revealed to participants, study investigators or staff. For more details see Hall et al., Nature Medicine, PMID: 33479499.
Blinding	Due to the nature of the diet interventions, once the food was delivered blinding of the subjects, investigators, or staff was not possible. However, all subjects were blinded to the primary and secondary aims of this study and were blinded to their data, including daily weight, glucose, and ketone measurements. For more details see Hall et al., Nature Medicine, PMID: 33479499.

Reporting for specific materials, systems and methods

We require information from authors about some types of materials, experimental systems and methods used in many studies. Here, indicate whether each material, system or method listed is relevant to your study. If you are not sure if a list item applies to your research, read the appropriate section before selecting a response.

Materials & experimental systems

n/a	Involved in the study
☐	☒ Antibodies
☒	☐ Eukaryotic cell lines
☒	☐ Palaeontology and archaeology
☒	☐ Animals and other organisms
☐	☒ Clinical data
☒	☐ Dual use research of concern

Methods

n/a	Involved in the study
☒	☐ ChIP-seq
☐	☒ Flow cytometry
☒	☐ MRI-based neuroimaging

Antibodies

Antibodies used	CD197 (BD Biosciences, BUV395, cat# custom, clone: 150503), Live/Dead stain (ThermoFisher, Live/Dead Blue, cat# L23105), CD16 (BD Biosciences, BUV496, cat# 612944, clone: 3G8), HLA-DR (BD Biosciences, BUV661, cat# 612980, clone: G46-6), CD196 (BD Biosciences, BUV737, cat# 564377, clone: 11A9), CD183/CXCR3 (BD Biosciences, BUV805, cat# 742048, clone: IC6/CXCR3), IgD (BD Biosciences, BV421, cat# 562518, clone: IA6-2), CD4 (BD Biosciences, eFlour450 (V450), cat# 560345, clone: SK3), CD127 (BD Biosciences, BV480, cat# 566101, clone: HIL-7R-M21), CD19 (BD Biosciences, BV570, cat# custom, clone: HIB19), CD194/CCR4 (Biolegend, BV605, cat# 359418, clone: 1G1), CD123 (BD Biosciences, BV650, cat# 563405, clone: 7G3), CD25 (BD Biosciences, BV711, cat# 563159, clone: 2A3), CD14 (BD Biosciences, BV750, cat# 746920, clone: M5E2), CD27 (BD Biosciences, BV786, cat# 563327, clone: L128), CD45RA (BD Biosciences, BB515, cat# 564552, clone: H100), CD38 (BD Biosciences, PerCP-Cy5.5, cat# 5514000, clone: HIT2), CD24 (BD Biosciences, BB700, cat# 566524, clone: ML5), CD45 (BD Biosciences, BB790, cat# custom, clone: HI30), CD8 (BD Biosciences, PE, cat# 555367, clone: RPA-T8), CD45RO (Beckman Coulter, PE-Texas Red, cat# IM2712U, clone: UCHL1), CD11c (BD Biosciences, Pe-Cy5, cat#: 551077, clone: B-Ly6), CD20 (ThermoFisher, Pe-Cy5.5, cat# MHCD2018, clone: HI47), CD185/CXCR5 (Biolegend, Pe-Cy7, cat# 356924, clone: RF8B2), CCR10 (R&D Systems, AlexaFlour 647 (APC), cat# FAB3478A, clone: 314305), CD56 (BD Biosciences, APC-R700, cat# 566139, clone: NCAM 16.2), CD3 (BD Biosciences, APC-H7, cat# 560176, clone: SK7)
Validation	All antibodies have been validated by the vendors. For BD Biosciences: The specificity is confirmed using multiple methodologies that may include a combination of flow cytometry, immunofluorescence, immunohistochemistry or western blot to test staining on a combination of primary cells, cell lines or transfectant models. All flow cytometry reagents are then titrated on the relevant positive and negative cell populations. ThermoFisher: The specificity of the antibody is validated by using a comprehensive approach that is tailored to the antibody target and the relevant application including Independent Antibody Verification by utilizing two independent antibodies for the same protein target that target nonoverlapping epitopes of an antigen and confirming similar results with multi-lysate western blots, IHC arrays, immunofluorescence of multiple cell lines, immunoprecipitation, flow cytometry, and other antibody applications. Biolegend: For quality control each lot of each antibody is quality control tested by immunofluorescent staining with flow cytometric analysis. Beckman Coulter: All antibodies are validated against the clinical standards CE-IVD and ASR. R&D Systems: Each antibody is manufactured manufactured under compliance with ISO 9001:2015 and/or ISO 13485:2016/MDSAP guidelines, undergoing rigorous quality control testing to ensure lot-to-lot consistency and outstanding performance. All antibodies are tested for cross-reactivity with closely related molecules using a variety of applications, including direct ELISA, to ensure specificity.

Clinical data

Policy information about clinical studies

All manuscripts should comply with the ICMJE guidelines for publication of clinical research and a completed CONSORT checklist must be included with all submissions.

Clinical trial registration	ClincalTrails.gov Identifier NCT03878108
Study protocol	The full protocol is available at the Open Science Framework website (https://osf.io/)

Data collection	The study was conducted from April of 2019 to March of 2020 at the Metabolic Clinical Research Unit of the NIH Clinical Center.
Outcomes	The first primary outcome compared the mean intake between each two-week diet period. The second primary outcome compared the mean energy intake on the second week of each diet period. These results were reported in Hall et al., Nature Medicine, PMID: 33479499. The primary exploratory aim of this study was to compare changes in immunity, microbiome composition and function, and metabolite profile between each two-week period of diet. These results are reported in this manuscript.

Flow Cytometry

Plots

Confirm that:

☒ The axis labels state the marker and fluorochrome used (e.g. CD4-FITC).

☒ The axis scales are clearly visible. Include numbers along axes only for bottom left plot of group (a 'group' is an analysis of identical markers).

☒ All plots are contour plots with outliers or pseudocolor plots.

☒ A numerical value for number of cells or percentage (with statistics) is provided.

Methodology

Sample preparation	PBMC from 21 samples were thawed and washed in RPMI containing 50U/ml benzonase nuclease then PBS. Cells were incubated with LIVE/DEAD Fixable Blue Dye (Life Technologies), washed and re-suspended in 100ul of FACS buffer (PBS with 0.5% fetal calf serum, 0.5% normal mouse serum and 0.02% NaN3), before incubation for 30 minutes with fluorochrome-conjugated antibodies. Cells were washed an additional two times with FACS buffer, fixed in 1% paraformaldehyde, and acquired using an Aurora spectral cytometer (Cytek Biosciences).
Instrument	Aurora spectral cytometer (Cytek Biosciences)
Software	Data collection was performed with the Aurora spectral cytometer software SpectroFlo version 2.2.0. For analysis FlowJo software version 10 (BD Biosciences) was used.
Cell population abundance	No cell population was sorted.
Gating strategy	Cell populations were gated and assessed based on previous reports (PMID: 29288606).

☒ Tick this box to confirm that a figure exemplifying the gating strategy is provided in the Supplementary Information.
